# The eubiotic perspective on utilization of tannins in phytotherapy and nutrition of pigs

**DOI:** 10.3389/fphar.2025.1649388

**Published:** 2025-08-25

**Authors:** Yuliia Kostenko, Inna Vlasova, Marcin Równicki, Philip Krüsselmann, Wilfried Vahjen, Jürgen Zentek, Aleksandra Tymoszewska, Jakub P. Piwowarski

**Affiliations:** ^1^ Microbiota Lab, Department of Pharmaceutical Microbiology and Bioanalysis, Medical University of Warsaw, Warsaw, Poland; ^2^ Institute of Animal Nutrition, Freie Universität Berlin, Berlin, Germany

**Keywords:** tannins, eubiotics, phytotherapy, gut homeostasis, post-weaning diarrhea

## Abstract

Gastrointestinal eubiosis is essential for maintaining overall host wellbeing. Post-weaning diarrhea (PWD) is a common issue in pig development, arising from weaning stress, which disrupts the gut microbiota balance and increases susceptibility to infections. The primary bacterial pathogen linked to PWD is enterotoxigenic *Escherichia coli* (ETEC). While antibiotics have traditionally been used for prevention and treatment of ETEC infections, their use is declining due to the emergence of multidrug-resistant pathogens and restrictions on the use of growth-promoting antimicrobials. Consequently, eubiotics are increasingly valued in pig nutrition as a safer alternative to antibiotics. While prebiotics and probiotics are well-studied, phytochemicals like tannins, despite the long history of their traditional use in ethnoveterinary medicine, remain largely unexplored. This review explores the eubiotic properties of tannins and their potential applications in swine nutrition and phytotherapy. *In vitro* and *in vivo* studies demonstrate that tannin-rich plant materials positively influence intestinal microbiota and epithelium, resulting in enhanced nutrient absorption, growth performance, and overall health in pigs. Moreover, they indicate that tannins possess antioxidant, anti-inflammatory, immunomodulatory, and antiparasitic properties which can be beneficial in pig farming. This review also highlights the safety of tannin supplementation, along with its environmental and economic advantages. Furthermore, it discusses potential strategies to mitigate tannin toxicity. Finally, it points out the existing research gaps and suggests directions for further research. In summary, it presents tannins as promising eubiotic agents for improving gut health and combating PWD.

## 1 Introduction

In recent years, the concept of eubiosis has emerged as a novel paradigm in animal health management. This approach represents a shift from suppressing the growth of pathogenic bacteria in gastrointestinal microbiota through antibiotics to actively supporting and modulating beneficial microbial populations and host immune system (Santovito et al., 2018; Mnisi et al., 2025). Eubiosis refers to the optimal microbial balance within the gastrointestinal tract, which is a critical factor for achieving optimal animal performance, nutrient utilization, and the development of a robust immune system. In this context, feed additives with scientifically proven efficacy can serve as valuable tools in promoting eubiosis and mitigating microbial imbalances within the gut environment, commonly referred to as dysbiosis (Clemente et al., 2012). These feed additives emerging from a concept of maintaining or rebalancing a microbial community in gastrointestinal tract, though very heterogenic in their chemical and biological characteristics, are collectively identified as eubiotics (Nowak et al., 2017; Santovito et al., 2018). Given the increasing restrictions on the routine use of antibiotics in livestock production, particularly for prophylactic and metaphylactic purposes, eubiotics are gaining importance as viable alternatives to support gut health and overall animal wellbeing.

Tannins, a group of large molecular weight compounds of plant origin, have gained attention as effective eubiotics with potential application in maintenance of gut microbiota balance (Molino et al., 2023). Tannins are polyphenolic secondary metabolites found in higher plants. They are divided into two main categories based on their chemical structure. The first group, includes galloyl esters and their derivatives, where galloyl units or related structures are bound to various core molecules such as polyols, catechins, or triterpenoids, which include gallotannins, ellagitannins, and complex tannins. The second group consists of oligomeric or polymeric proanthocyanidins, referred to as condensed tannins, which are characterized by diverse patterns of interflavanyl bonding and substitutions (Khanbabaee and van Ree, 2001). The traditional use of tannins-containing medicinal plants in various cultures across the globe is consequently reported by contemporary ethnobotanical and ethnopharmacological studies as well as numerous historical sources (Kiss and Piwowarski, 2018; Ren et al., 2021; Li et al., 2022). Ethnoveterinary studies conducted in different regions of the world consistently report - plant materials containing different types of tannins as traditional remedies applied in gastrointestinal tract-related ailments of domesticated animals (Mertenat et al., 2020; Schlittenlacher et al., 2022; Munengwa et al., 2025).

Taking into consideration the traditional knowledge and currently conducted studies on utilization of tannin-containing plant materials in maintenance of gut health of pigs, this review aims to go beyond the common reception of tannins as strong and unselective protein binding molecules acting as astringents (Haslam, 1996), and look for their therapeutic and preventive potential from the perspective of eubiosis maintenance in gastrointestinal tract of pigs. By integrating insights from traditional practices and contemporary research, this work aims to present a more nuanced and evidence-based evaluation of tannins, not as mere anti-nutrients, but as potential allies in promoting sustainable and antibiotic-free livestock production through the support of gut health in pigs.

## 2 Gastrointestinal dysbiosis in pigs

Dysbiosis, understood as an imbalance in intestinal microbiota, is a common issue in modern pig production. Several factors can lead to dysbiosis including stress, infections or antibiotic use. Among these, stress is particularly relevant, arising from challenges such as weaning, transportation, climate change, or suboptimal husbandry conditions. Weaning, which involves separation from the sow and transition from milk to solid feed, is one of the most stressful periods for piglets. During this critical time, numerous environmental and psychological stressors activate the hypothalamic-pituitary-adrenal axis, leading to cortisol release, which disrupts intestinal barrier integrity by downregulating tight junction proteins and increasing permeability (Molotla-Torres et al., 2023). This disruption allows bacterial translocation and subsequent immune activation, fueling a cycle of dysbiosis and gut inflammation (Geng et al., 2020). Furthermore, bidirectional signaling along the gut-brain axis, *via* neural, endocrine, and immune pathways, conveys stress induced changes in the intestine back to central nervous system, exacerbating microbial imbalance and barrier dysfunction (Nie et al., 2024). As a result, young piglets become more susceptible to gastrointestinal infections (Xiong et al., 2019).

Post-weaning diarrhea (PWD) is one of the most economically important diseases in pig farming. Its etiology is complex, involving multiple contributing factors, but it is frequently linked to infections caused by enterotoxigenic *Escherichia coli* (ETEC) (Rhouma et al., 2017). While antibiotics remain a cornerstone of infections treatment in swine production, their use can even further disrupt microbiota homeostasis causing intestinal dysbiosis and long-term adverse effects (Duan et al., 2022).

The administration of antibiotics in swine nutrition underwent significant changes following the adoption of Regulation (EC) No 1831/2003 of the European Parliament and of the Council. Before this regulation, antibiotics were commonly used in swine production not only therapeutically (to treat animals showing clinical symptoms) and prophylactically (to prevent disease outbreaks in healthy animals), but also as growth promoters, with sub-therapeutic doses frequently added to feed to improve weight gain and feed efficiency (Luecke et al., 1951). This widespread use contributed to the emergence of antibiotic-resistant bacteria, with resistance genes potentially transferring to human pathogens (Giurazza et al., 2021). In response, regulation (EC) No 1831/2003 established a legal framework for the use of feed additives, leading to a full ban on antibiotics as growth promoters in the EU as of 1 January 2006. While prophylactic use was still allowed under certain conditions, it has since been increasingly restricted, especially with the implementation of Regulation (EU) 2019/6, effective from 2022, which limits prophylactic administration to exceptional cases under strict veterinary oversight. Therapeutic use remains permitted but is also tightly regulated by veterinary prescription.

Until recently, pharmacological doses of zinc oxide (ZnO) were commonly used in pig nutrition, especially for weaned piglets. ZnO can support gut health and reduce the risk of PWD through several mechanisms including modulation of gut microbiota, direct anti-microbial activity, improvement of intestinal barrier function, and anti-inflammatory effects. ZnO modulates gut microbiota by suppressing harmful bacteria such as *E*. *coli* and supporting the growth of beneficial bacteria like *Lactobacillus*, a natural component of the piglet gut microbiota. This microbial shift is partly facilitated by ZnO’s ability to lower intestinal pH through reduced ammonia production, creating an environment less favorable for pathogens such as *E. coli*, and more suitable for acid-tolerant bacteria, such as *Lactobacillus*. Additionally, ZnO can directly damage bacterial cell membranes or inhibit essential metabolic pathways, thereby inhibiting pathogen growth. It also strengthens the intestinal barrier by upregulating tight junction proteins, reducing gut permeability. Finally, ZnO reduces inflammation by downregulating pro-inflammatory cytokines, helping to maintain gut integrity during weaning stress (Tang et al., 2024). However, its usage has also raised concerns due to environmental impact, mainly the accumulation of zinc in manure. This results from its low absorption in the piglet’s gut, with most of the zinc being excreted and potentially polluting soil and water. There is also an increasing evidence that its use can contribute to the development of bacterial resistance to both heavy metals and antibiotics (Bednorz et al., 2013). As a result, the use of medicinal levels of ZnO has been banned in the EU since 2022 (Regulation 2019/6). This regulatory change prompted the urgent need for alternative strategies to prevent PWD.

One of the most promising solutions to improve gut microbiota balance and prevent PWD is the use of eubiotics, especially tannins (Nowak et al., 2017). Historical records highlight the long-standing use of tannins for their anti-diarrheal properties in both human and veterinary medicine dating back to ancient times, long before the discovery of antibiotics (Piwowarski et al., 2015). Renewed scientific interest in tannins has highlighted their potential as natural feed additives capable of supporting intestinal health and reducing reliance on synthetic antibiotics and metal-based supplements. Several studies have demonstrated that, when used at appropriate dose and duration, tannins do not cause adverse effects or long-term accumulation in tissues (Espín et al., 2007; Caprarulo et al., 2020; Nuamah et al., 2024). Moreover, their inclusion in pig diets has no significant impact on the chemical or amino acid composition of adipose tissue (Bottegal et al., 2024) or liver tissue (Bilić-Šobot et al., 2016), and has only minor effects on carcass value of fattening pigs (Bottegal et al., 2024). Consequently, low-dose tannin supplementation does not negatively affect meat quality (Bilić-Šobot et al., 2016; Bottegal et al., 2024), supporting the safety of pork for human consumption. These findings reinforce the acceptability of tannins as functional feed additives in pig diets, as long as their use is carefully managed.

Importantly, tannins are primarily derived from natural sources and undergo aerobic decomposition within weeks, preventing their accumulation in the environment and minimizing their impact on ecosystems. Unlike synthetic antibiotics and chemical additives, tannins do not persist or disrupt ecological balance. Their low toxicity and biodegradability minimize the risks associated with environmental exposure, making them a safe long-term solution in livestock management (Sharma et al., 2021). Although tannins play a significant role in modulating microbial populations in pigs, their rapid biodegradability also lowers the risk of contributing to the development and spread of resistant bacteria. By avoiding prolonged selective pressure in the environment, tannins are less likely to facilitate the emergence of resistant strains, making them a safer and more sustainable alternative in the context of antimicrobial resistance (Girard and Bee, 2020).

Livestock production releases harmful chemicals into the environment, including methane (CH_4_) and ammonia (NH_3_), which pose risks to both humans and ecosystems. Recent research highlights the potential of tannin-containing medicinal plants, to reduce these emissions. Tannins inhibit methanogens, lowering methane output, and improve nutrient digestibility, decreasing undigested material available for fermentation in manure (Hossain et al., 2024). Additionally, tannins lower ammonia concentrations, a toxic compound that can impair pig performance at high levels. Tannins can reduce ammonia formation by increasing the activity of digestive enzymes, as well as by suppressing the growth of proteolytic bacteria responsible for producing ammonia. This dual action helps to reduce the environmental burden associated with livestock waste (Hossain et al., 2024). Interestingly, some studies consider incorporating tannins into feed strategies as an alternative approach to mitigate pork odor (Bee et al., 2017; Bahelka et al., 2023). Tannins in swine diets can reduce skatole accumulation in adipose tissue, a compound responsible for unpleasant pork odors. This reduction can not only improve meat quality but also minimize environmental odor emissions (Bahelka et al., 2023).

Economically, tannins are considered cost-effective due to their natural abundance, wide availability, and relatively low extraction and production costs. Their incorporation into pig diets has been associated with a reduction in PWD which not only lowers the need for medical treatments and veterinary interventions but also minimizes production losses linked to high mortality rates in affected animals. By promoting better gut health and enhancing feed efficiency, tannins also contribute to improved weight gain and overall animal productivity (Girard and Bee, 2020).

In the following sections, we explore current research on the use of tannins in pig nutrition, covering both *in vitro* and *in vivo* studies. Particular attention is given to their antimicrobial, microbiota-modulating, and epithelial-modulating activities. Strategies for optimizing tannin application in swine production are then discussed, with the aim of minimizing their potential toxicity. Finally, future perspectives and research directions are presented.

## 3 Health benefits of tannins from *in vitro* studies

### 3.1 Impact of tannins on the growth of PWD-associated pathogens

*Escherichia coli*, particularly ETEC, are the primary pathogen associated with PWD. However, other bacterial species, including *Salmonella* spp. and *Clostridium perfringens* have also been implicated in the onset and progression of PWD. These pathogens can act individually or synergistically, further exacerbating the severity of the disease. Importantly, numerous *in vitro* studies have demonstrated that the growth of these bacterial strains is significantly diminished in the presence of tannins. Table 1 summarizes the effects of tannins extracted from various plant sources on bacterial strains commonly associated with PWD in piglets.

**TABLE 1:** Antibacterial properties of tannins form *in vitro* studies.

Source	Form	Tannin content	Type of tannin	Dose and time of incubation/MIC/MBC	Tested bacterial strains	References
*Muntingia calabura* L., leaves	ethanolic extract	0.0655 mg TAE/g d.w	HT	12.5%–75% extract, 24 h	*E. coli, S.* Typhimurium	Gurning et al. (2021)
Grape seeds (*Vitis* spp.)	proanthocyanidins (commercial product)	purity >96%	CT	>10 mg/mL >10 mg/mL	*E.* *coli* CMCC 44102 *S.* Typhimurium CMCC 50094	Ding et al. (2021)
	proanthocyanidins (PC)-loaded chitosan (CH) nanoparticles	1:0.5 CH:PC ratio		0.625 mg/mL 2.5 mg/mL	*E. coli* CMCC 44102 *S.* Typhimurium CMCC 50094	
Purple loosestrife flowers (*Lythrum salicaria* L.)	castalagin, vescalagin	—	HT	0.5 mM, 24 h	*E. coli* (castalagin) *C. perfringens*	Puljula et al. (2020)
English oak acorns *(Quercus* spp.*)*	vescavaloninic acid	—	HT	0.5 mM, 24 h	*E. coli* *C. perfringens*	
Sea buckthorn leaves *(Hippophae rhamnoides)*	stachyurin, casuarinin hippophaenin B	—	HT	0.5 mM, 24 h	*E. coli* *C. perfringens*	
Meadowsweet inflorescence *(Filipendula ulmaria)*	tellimagrandin I, tellimagrandin II	—	HT	0.5 mM, 24 h	*E. coli* *C. perfringens*	
Raspberry leaves *(Rubus idaeus)*	pedunculagin	—	HT	0.5 mM, 24 h	*C. perfringens*	
Sea buckthorn leaves	casuarictin, strictinin	—	HT	0.5 mM, 24 h	*E. coli* (casuarictin) *C. perfringens* (strictinin)	
Wood cranesbill leaves and flowers *(Hippophae rhamnoides)*	geraniin	—	HT	0.5 mM, 24 h	*E. coli*	
*T. chebula* fruits	punicalagin	—	HT	0.5 mM, 24 h	*E. coli* *C. perfringens*	
Raspberry leaves *(Rubus idaeus)*	sanguiin H-6, lambertianin C	—	HT	0.5 mM, 24 h	*E. coli* *C. perfringens*	
Meadowsweet inflorescence *(Filipendula ulmaria)*	rugosins D and E	—	HT	0.5 mM, 24 h	*E. coli* *C. perfringens* (rugosin D)	
Silverweed leaves *(Potentilla anserine)*	agrimoniin	—	HT	0.5 mM, 24 h	*E. coli* *C. perfringens*	
Purple loosestrife flowers and leaves (*Lythrum salicaria* L.)	salicarinin A	—	HT	0.5 mM, 24 h	*E. coli* *C. perfringens*	
TA	pentagalloylglucose	—	HT	0.5 mM, 24 h	*E. coli* *C. perfringens*	
Purple loosestrife (*Lythrum salicaria* L.)	aqueous extract	Castalagin 33.1 mg/g, vescalagin 47.5 mg/g, salicarinin A 52.1 mg/g, salicarinin B 45.0 mg/g	HT	MIC = 2 mg/mL	*E. coli* IMT 0147:K89:K88 *E. coli* DSM 2840	Granica et al. (2020)
	Castalagin	—	HT	MIC = 0.5 mg/mL	*E. coli* IMT 0147:K89:K88 *E. coli* DSM 2840	
Chestnut (*Castanea sativa* Mill.)	aqueous extract	75 g of tannin/100 g of dry matter	HT	MIC = 7 mg/mL MBC_F4+_ = 9 mg/mL MBC_F18+_ = 8 mg/mL	ETEC strains, harboring F4 (F4+) and F18 (F18+) adhesive fimbriae	Reggi et al. (2020)
Quebracho *(Schinopsis* spp.)	aqueous extract	75 g of tannin/100 g of dry matter	CT	MIC = 6 mg/mL MBC_F4+_ = 9 mg/mL MBC_F18+_ = 8 mg/mL		
Chestnut and quebracho (*Castanea sativa* Mill., *Schinopsis* spp.)	aqueous extract	75 g of tannin/100 g of dry matter	HT and CT	MIC = 6 mg/mL MBC = 8 mg/mL		
root of *Rosa roxburghii* Tratt	strictinin isomers	—	HT	MIC = 0.125 mg/mL	*E. coli* ATCC 25922	Ma et al. (2020)
*Miconia latecrenata* leaves	aqueous extract	82.89 ± 2.59 μg TAE/mg, mailny isomers of ellagitannins 1,2,3,5-tris-galloyl-4,6-HHDP-glucose	HT	7–8 mg/mL	*E. coli* clinical isolate	Gontijo et al. (2019)
American cranberry (*Vaccinium macrocarpon*)	proanthocyanidins powder (commercial product)T	—	CT	MIC = 18 mg/mL MBC = 36 mg/mL	EPEC and ETEC	Alshaibani et al. (2017)
*Acacia mearnsii* De Wild., bark	Weibull AQ (commercial product)	—	CT	MIC = 20 mg/mL MBC = 20 mg/mL	*E. coli* ATCC 25922	Klug et al. (2017)
	Tanfloc SG (commercial product)	—	CT	MIC = 5 mg/mL MBC = 10 mg/mL	*E. coli* ATCC 25922	
	Tanfloc SM (commercial product)	—	CT	MIC = 5 mg/mL MBC = 10 mg/mL	*E. coli* ATCC 25922	
laurel wood *(Laurus nobilis)*	cinnamtannin B-1	purity of 95%	CT	MIC = 1 mg/mL	*E. coli* CCUG 47553*, E. coli* CCUG 47557	Alejo-Armijo et al. (2017)
	procyanidin B-2	purity of 93%	CT	MIC = 1 mg/mL	*E. coli* CCUG 47553*, E. coli* CCUG 47557	
*Phyllanthus muellerianus*, aerial part	aqueous extract	4.3% w/w grraniin	HT	MIC = 5 mg/mL; MBC = 50 mg/mL	*E. coli* ATCC 25922	Boakye (2016)
	geraniin	96% w/w	HT	MIC = 1.25 mg/mL; MBC = 10 mg/mL	*E. coli* ATCC 25922	
*Erythrophleum guineensis* stem barks	Ethanol-water 70/30 extract	0.865 mg EC/mL	CT	MIC = 1.25 mg/mL MBC = 5 mg/mL	*S. enterica* NR 13555 *E. coli* ATCC 25922	Mirelle et al. (2016)
*Caesalpinia spinosa* pods, *Rhus semialata* and *Rhus typhina* gallnuts, *Quercus infectoria* and *Rhus coriaria* leaves	TA (commercial product)	—	HT	MIC = 1.88 mg/mL MIC = 6.25 mg/mL MIC = 5 mg/mL	*E. coli* O157:H7 *S*. Enteriditis *S*. Typhimurium	Widsten et al. (2014)
	TA (commercial product)	—	HT	MIC = 1.88 mg/mL MIC = 2.5 mg/mL MIC = 3.75 mg/mL	*E. coli* O157:H7 *S*. Enteriditis *S*. Typhimurium	
*Acacia* spp. bark	mimosa tannin (commercial product)	—	CT	MIC = 7.50 mg/mL MIC = 6.25 mg/mL MIC = 20 mg/mL	*E. coli* O157:H7 *S*. Enteriditis *S*. Typhimurium	
Pomegranate *(Punica granatum *L.*)*	punicalagin	—	HT	MIC = 0.5 mg/mL MIC = 0.25–1 mg/mL	*S.* Typhimurium SL1344 *Salmonella* isolates from raw chicken	Li et al. (2014)
*Caesalpinia spinosa* (Molina) *Kuntze*	aqueous extract	Gallotannins 34.4 mg GAE/mL, free gallic acid 2.4 mg GA/mL	HT	MIC = 4.25 mg GAE/mL	*S.* Enteriditis	Aguilar-Galvez et al. (2014)
	Hydrolysate of aqueous extract (4 h)	Gallotannins 19.4 mg GAE/mL, free gallic acid 17.3 mg GA/mL	HT	MIC = 1.46 mg GAE/mL	*E. coli*	
Guava leaves (*Psidium guajava* L)	Extract with 30% ethanol	2.351 mg/g	CT	20% and 30% extract	*E. coli*	Mailoa et al. (2014)
*Terminalia chebula* fruits	1,2,6-tri-O-galloyl-β-D-glucopyranose	—	HT	MIC = 0.0121 mg/mL	*E. coli* ATCC 8739	Bag et al. (2013)
*Rhus semialata*	TA (commercial product)	—	HT	0.4 mg/mL, 24 h	*E. coli* serotype O157:H7 strains	Wang et al. (2013)
Purple prairie clover (*Dalea purpurea* Vent)	extracted with a 70% acetone solution	—	CT	0.2 mg/mL	*E. coli* serotype O157:H7 strains	
—	Corilagin (commercial product)	purity > 99%	HT	MIC = 0.0625 mg/mL	*E*. *coli* ATCC 8739	Li et al. (2013)
Mango Kernels (*Mangifera indica* L.)	Hepta-O-galloylglucose	—	HT	MIC = 0.6–0.8 mg/mL, MBC = 0.8–2.2 mg/mL MIC = 0.7 mg/mL, MBC = 2.8 mg/mL	*E. coli* AW 1.7, *E. coli* TMW 2.497, ETEC ECL 13086, ETEC ECL 13795, ETEC ECL 13998, ETEC ECL 14408, *E. coli* FUA 1062 (SLT1), *E. coli* FUA 1064 (SLT2) *S.* Typhimurium	Engels et al. (2011)
Chestnut wood (*Castanea sativa* Mill.)	commercial extracts	75.2%	HT	1, 3, 6 mg/mL (extract), 24 h 1, 3, 6 mg/mL (fraction), 24 h	*S.* Typhimurium SL1344nal^r^	Costabile et al. (2011)
Chestnut wood (*Castanea sativa* Mill.)		91.6%	HT	1, 3, 6 mg/mL (extract), 24 h 1 and 6 mg/mL (fraction), 24 h		
Tara (*Caesalpinia spinosa*)		95.1%	HT	1, 3, 6 mg/mL (extract), 24 h 1, 3, 6 mg/mL (fraction), 24 h		
Chinese galls (sumach) (*Rhus coriaria)*		94.0%	HT	1, 3, 6 mg/mL (extract), 24 h 1, 3, 6 mg/mL (fraction), 24 h		
Quebracho (*Schinopsis* spp.)		77.5%	CT	1 and 6 mg/mL (fraction), 24 h		
Quebracho Tannino QS-SOL (*Schinopsis* spp.)		92.1%	CT	1, 3, 6 mg/mL (fraction), 24 h		
San Ramón (*Calliandra calothyrsus*)	isolated fractions	-	CT	6 mg/mL, 24 h		
Patulul (*Calliandra calothyrsus*)	isolated fractions	-	CT	3, 6 mg/mL, 24 h		
Sweet chestnut wood (*Castanea sativa* Mill.)	Globatan (commercial product)	75% tannins	HT	MIC = 160 μg/mL (46 strains) MIC = 320 μg/mL (11 strains)	*S.* Typhimurium, 57 isolates from pigs	Van Parys et al. (2010)
				Growth reduction 25–50 μg/mL Growth inhibition 100 μg/mL MBC = 500 μg/mL	*S.* Typhimurium strain 112910a	

HT, hydrolysable tannins; CT, condensed tannins.

The inhibition of *E. coli* growth was reported in the presence of significant number of hydrolysable tannins (HT), including plant extracts and isolated compounds. For example, extracts of *Lythrum salicaria* and *Phyllanthus muellerianus* showed antibacterial activity, as did their isolated compounds, castalagin and geraniin, respectively (Boakye, 2016; Granica et al., 2020). Similarly, 1,2,6-tri-*O*-galloyl-β-D-glucopyranose, a compound isolated from *Terminalia chebula* fruits extract, was active against *E. coli* (Bag et al., 2013). Antimicrobial effects were also demonstrated for extracts of *Castanea sativa* (Reggi et al., 2020), *Miconia latecrenata* (Gontijo et al., 2019), *Rosa roxburghii* root (Ma et al., 2020), and a hydrolysate of *Caesalpinia spinosa* extract (Aguilar-Galvez et al., 2014). Furthermore, HT-rich commercial products such as corilagin and tannic acid (TA) also showed inhibitory effects (Li et al., 2013; Wang et al., 2013). Comparable antibacterial activity against *E. coli* was observed with condensed tannins (CT) (Wang et al., 2013; Alejo-Armijo et al., 2017; Klug et al., 2017). For instance, chestnut and quebracho tannins were effective in inhibiting the most relevant *E. coli* pathotypes associated with PWD—those expressing F4+ and F18+ adhesive fimbriae (Reggi et al., 2020). Moreover, proanthocyanidins from American cranberry demonstrated activity against EPEC and ETEC (Alshaibani et al., 2017). Finally, tannin-rich extract from guava leaves (*Psidium guajava* L.) displayed antimicrobial properties against *E. coli* (Mailoa et al., 2014).

The inhibition of *Salmonella* spp. growth was also observed in the presence of various tannin sources. Punicalagin, HT from pomegranate, demonstrated antibacterial properties against multiple *Salmonella* isolates from raw chicken as well as *S.* Typhimurium reference strain SL1344 (Li et al., 2014). This bacterial strain was also inhibited by HT from chestnut wood, tara, and Chinese galls extracts, as well as by CT from quebracho extract, and from isolated fractions of *Calliandra calothyrsus* (Costabile et al., 2011). Finally, sweet chestnut wood extract Globatan had inhibitory effect on *S.* Typhimurium isolates from pigs and *S.* Typhimurium strain 112910a, which is a field isolate commonly used in studies on gastrointestinal infections in swine (Van Parys et al., 2010).

Tannins are associated with a broad spectrum of antimicrobial activity, and numerous studies have demonstrated their simultaneous effectiveness against several PWD-linked pathogens. Plant extracts from *Erythrophleum guineensis* stem barks, and *Muntingia calabura* leaves showed inhibitory activity against not only *E. coli*, but also *Salmonella* spp. (Mirelle et al., 2016; Gurning et al., 2021). An inhibitory activity against *E. coli* and *S.* Typhimurium was observed for proanthocyanidins derived from grape seeds (Ding et al., 2021) and Hepta-O-galloylglucose (HT) from Mango Kernels (*Mangifera indica* L.) (Engels et al., 2011). An inhibitory activity against *E. coli* and *S.* Enteritidis was observed for HT from *C. spinosa* extract (Aguilar-Galvez et al., 2014). The growth of *S.* Enteritidis, *S.* Typhimurium and *E. coli* was reduced in the presence of TA and mimosa tannins (Widsten et al., 2014). Finally, HT from various plant sources were found to inhibit *E. coli* and *C. perfringens* (Puljula et al., 2020).

Tannins exhibit antimicrobial activity through various mechanisms, including the disruption of bacterial membranes (Li et al., 2013; Wang et al., 2013; Alshaibani et al., 2017). Tannins can also chelate essential metal ions such as iron and zinc, which are crucial for bacterial metabolism, and bind to bacterial proteins and enzymes, interfering with their function (Ma et al., 2020). These changes inhibit bacterial growth and viability. Tannins also decreased bacterial swimming and swarming motility *via* downregulation of the motility-related genes of *Salmonella* (Li et al., 2014). These findings highlight tannins’ potential as natural antimicrobial agents in addressing the multifactorial causes of PWD. However, many studies do not evaluate the mechanisms of action in detail, warranting further investigation.

### 3.2 Impact of tannins on virulence factors of PWD-associated pathogens

Although the exact mechanisms of ETEC pathogenesis are not fully understood, it is known that it binds specifically to receptors on the surface of the intestinal epithelial cells (Brosnahan and Brown, 2012) and releases toxins–either heat-stable or heat-labile–that disrupt gut integrity, trigger inflammation, promote fluid secretion, and ultimately cause diarrhea (Heo et al., 2013; Kim et al., 2022). Multiple studies have reported that tannins not only inhibit the growth of bacteria implicated in PWD, but also interfere with key virulence factors, such as bacterial adhesion, toxin production and binding, and biofilm formation, thereby limiting the pathogenic potential of these microorganisms.

The adhesion of *E. coli* to receptors on intestinal epithelial cells is a critical step in the infection process (Heo et al., 2013; Kim et al., 2022). Notably, tannins have demonstrated the ability to inhibit bacterial adhesion to the IPEC-J2 cell line, an *in vitro* model derived from porcine jejunal epithelial cells, commonly used to study the effects of tannins on intestinal health in pigs. Specifically, water extract from *L. salicaria* L. containing C-glycosylic ellagitannins and isolated castalagin significantly reduced enteropathogenic *E. coli* (EPEC) adhesion by 20.4% and 37.7%, respectively (Granica et al., 2020). Importantly, similar effects were observed in *ex vivo* studies. Polyphenol extract from cocoa beans, pentagalloyl glucose crude and purified HT successfully inhibited ETEC adhesion to pig intestinal brush borders (Verhelst et al., 2010). Moreover, cranberry extract prevented the adherence of F4^+^ and F18^+^ *E. coli* to porcine intestinal villi without reducing bacterial viability (Coddens et al., 2017). These findings suggest that tannins could play an important role in preventing intestinal infections by limiting bacterial adhesion, highlighting their potential as therapeutic agents for improving intestinal health in pigs.

Tannins may also effectively interact with toxins released by ETEC strains after adhesion. Pentagalloyl glucose crude and purified HT inhibited the binding of the heat-labile toxin produced by ETEC (Verhelst et al., 2010). Later pentagalloylglucose significantly inhibited the binding of the heat-labile toxin to the intestinal receptor GM1, potentially due to the formation of large LT–polyphenol aggregates (Verhelst et al., 2013). The findings suggest that tannins’ distinctive composition may play a key role in reducing toxin-mediated damage during PWD in pigs.

Biofilm formation is a crucial virulence factor in bacteria, playing a key role in the development of chronic, drug-resistant infections. The multifactorial nature and complex structure of biofilms make them particularly challenging to treat (Koo et al., 2017). Studies have shown that ellagic acid and TA effectively reduce biofilm formation in *E. coli* F18 (Hancock et al., 2010). TA has also demonstrated synergy with other antibacterial agents. For instance, nanocomposites of TA and silver have shown potent anti-bacterial and anti-biofilm properties. TA likely exerts its effects by inhibiting quorum sensing—a regulatory process controlling bacterial behaviors such as biofilm formation (Liu L. et al., 2020). Tannins capacity to inhibit biofilm formation renders them a promising class of compounds for the development of alternative treatments against pathogens, including those associated with PWD.

### 3.3 Modification of intestinal epithelial cells

The IPEC-J2 cell line is commonly used as a model to study the effects of tannins on intestinal epithelium. Table 2 summarizes research findings on the influence of tannins on epithelial health and monolayer integrity. Overall, the studies suggest that tannins exert a dose-dependent effect on IPEC-J2 cells viability and proliferation. While higher concentrations may impair cells viability, lower doses have been shown to exert protective or even stimulatory effects (Caprarulo et al., 2020). For instance, TA was found to decrease IPEC-J2 cells viability through several mechanisms, including oxidative stress induction, DNA damage, mitochondrial dysfunction, and the activation of the mitochondrial apoptotic pathway. It also caused S phase arrest, leading to cytotoxicity and reduced cell proliferation (Wang et al., 2019). However, subsequent findings by the same author showed that TA increased cell viability at certain concentrations, suggesting that while high doses or prolonged exposure have adverse effects, appropriate doses may confer benefits (Wang et al., 2022). Similarly, studies with *in vitro* digested chestnut and quebracho tannins revealed that high concentrations lead to a reduction in cell viability, while lower dosages have a beneficial effect, promoting cell survival (Reggi et al., 2020). Apart from survival ability of cells, there are some investigations focusing on tannins’ effect on growth and division of cells. For example, HT from chestnut and oak were found to accelerate proliferation of IPEC-J2 cell line but the mechanism of this action is unclear (Brus et al., 2013c). Taken together, these findings highlight the complex, dose-dependent effects of tannins on the viability of intestinal epithelial cells.

**TABLE 2:** Effects of tannins on intestinal epithelial cells from *in vitro* studies.

Source	Form	Tannin content	Type of tannin^a^	Dose and time of incubation	Effect	Mechanism of action^b^	References
—	TA (commercial product)	95% purity	HT	6.4 μM, 24 h pre-incubation, 2 h incubation with ETEC (multiplicities of infection (MOI) = 100)	Increased cells viability Improve in intestinal epithelial layer	↑expression of ZO-1, occludin and claudin-1 at both mRNA and protein levels	Liu and Guo (2024)
Chinese gallnut (*Galla Chinensis*)	TA (commercial product)	—	HT	0.5 and 12.5 µM, 3 h	Increased cells viability		Wang et al. (2022)
				2.5 and 5 µM, 18 h 2.5 and 5 μM, 12 h pre-incubation, 200 µM TBH 3 h incubation	Improve in intestinal epithelial layer	2.5 μM: ↑ mRNA abund. of ZO-1; 2.5 and 5 μM: ↑ mRNA abund. of ocludin, ↑ protein abund. of CLDN-1 and occludin	
-	Gallic acid	—	—	50 μmol/L, 24 h	Decrease of monolayer integrity	↓ protein abund. of CLDN-1	Tretola et al. (2021)
Purple loosestrife (*Lythrum salicaria* L.)	Water extract	Castalagin 33.1 mg/g, vescalagin 47.5 mg/g, salicarinin A 52.1 mg/g, salicarinin B 45.0 mg/g	HT	100 μg/mL, 24 h pre-incubation, infection with EPEC (MOI of 100, equivalent to 1 × 10^7^ bacteria per well.)	Stimulation of monolayer formation	↑ claudin 4 and ZO-1 protein production	Granica et al. (2020)
	Castalagin, vescalagin	—	HT	20 μM, 6 days	Stimulation of monolayer formation	No changes in claudin 4 and ZO-1 protein production	
	salicarinin A, salicarinin B	—	HT	20 μM, 6 days	Stimulation of monolayer formation	↑ claudin 4 production	
Chestnut and Quebracho 1:1 (*Castanea sativa* Mill., *Schinopsis* spp.)	Experimental diet (Plurimix, Fabermatica, CR, Italy) with 1.25% extract from chestnut and quebracho trees (commercial product), *in vitro* digested	2.91 g TAE/kg	HT, CT	0.33–21.33 mg/mL, 3 h	1.33 mg/mL – promotion of cells viability 5.32 mg/mL – reduction of cells viability	Not studied	Caprarulo et al. (2020)
Chestnut (*Castanea sativa* Mill.)	Hot water extract, *in vitro* digested	75% tannins	HT	50 and 200 μg/mL, 3 h 100 μg/mL, 3 h 400, 600, 800, 1,200 μg/mL, 3 h	Increased cells viability Unaltered cells viability Reduced cells viability	Not studied	Reggi et al. (2020)
Quebracho *(Schinopsis* spp.)	Hot water extract*,* *in vitro* digested	75% tannins	CT				
Chestnut and Quebracho 1:1 (*Castanea sativa* Mill., *Schinopsis* spp.)	Hot water extract, *in vitro* digested	75% tannins	HT, CT	50–200, 400 μg/mL, 3 h 600–1,200 μg/mL, 3 h 200–1,200 μg/mL, 3 h pre-incubation 50–400 μg/mL, 3 h pre-incubation	Increased cells viability Reduced cells viability Counteracted H_2_O_2-_induced stress by increasing cells viability Counteracted DSS-induced stress by increasing cells viability	Not studied	
-	TA (commercial product)	—	HT	10, 20, 40, 80 μM, 24 h	Decreased cells viability, dose dependent effect	Induced oxidative stress, DNA damage, mitochondrial dysfunction, mitochondrial pathway of apoptosis, S phase arrest	Wang et al. (2019)
Chestnut (*Castanea sativa* Mill.)	Gallic acid (commercial product)	—	HT	1–2 μg/mL, 5–10 days	Increased cellular proliferation	Not studied	Brus et al. (2013b)
Chestnut (*Castanea sativa* Mill.)	Farmatan (commercial product)	20.00 mg/g GAE DW	HT	1–31.25 μg/mL, 5–10 days	Increased cellular proliferation	Not studied	Brus et al. (2013c)
Oak *(Quercus* spp.*)*	Contan (commercial product)	13.60 mg/g GAE DW	HT	1–62.5 μg/mL, 5–10 days	Increased cellular proliferation	Not studied	Brus et al. (2013c)

^a^ HT, hydrolysable tannins; CT, condensed tannins.

^b^ ↑, increase; ↓, decrease.

The measurement of monolayer formation is also an effective parameter for assessing the impact of external agents, such as tannins, on cells. Tight junction proteins, such as claudins (CLDN), occludin (OCLN), zonula occludens proteins (ZO-1, ZO-2, ZO-3), are essential for forming a functional monolayer, providing both the permeability barrier and the cohesive cell-cell adhesion. In most *in vitro* studies, tannins have been shown to have a positive influence on the expression of tight junction proteins. Purple loosestrife (*L. salicaria* L.) water extract, along with its isolated ellagitannins—castalagin, vescalagin, salicarinins A and B—stimulated the formation of an IPEC-J2 monolayer. The extract was found to enhance the expression of CLDN-4 and ZO-1 proteins, whereas salicarinins A and B demonstrated the ability to upregulate CLDN-4 (Granica et al., 2020). Similarly, TA has been reported to upregulate the mRNA expression of OCLN and ZO-1 along with increasing protein levels of CLDN-1 and OCLN in cells with oxidative damage induced by tert-butyl hydroperoxide (TBH) (Wang et al., 2022). The other study also confirms that TA increased expression of OCLN, CLDN-1, ZO-1 at both mRNA and protein levels in the intestinal cells injured by *E. coli* (Liu and Guo, 2024). However, gallic acid has shown mixed results; it reduced monolayer integrity by decreasing CLDN-1 protein expression, although the lower concentrations used in the experiment had no negative effect on tight junction protein abundance (Tretola et al., 2021). These studies show that tannins, at appropriate concentrations, can support the formation of the cell monolayer.

### 3.4 Antioxidant, anti-inflammatory and antiparasitic properties of tannins *in vitro*

Tannins have been widely studied for their antioxidant properties and potential to mitigate oxidative stress in various IPEC-J2 models. In a model of oxidative stress induced by TBH, TA significantly mitigated the reduction in cell viability and improved cell morphology, highlighting its protective role in maintaining cellular health. The study showed that TA treatment reduces levels of reactive oxygen species and malondialdehyde (MDA), a product of lipid peroxidation, while boosting antioxidant markers like total antioxidant capacity, glutathione, and total glutathione (Wang et al., 2022). Similarly, TA was found to effectively counteract the oxidative stress caused by ETEC infection by increasing cell viability, enhancing the activity of antioxidant enzymes, and reducing MDA levels (Liu and Guo, 2024). The protective mechanism of TA may involve activation of the p62-Keap1-Nrf2 pathway, which plays a crucial role in regulating the cellular antioxidant response. This is supported by the observed increase in Nrf2 and GPX4 protein levels—both associated with enhanced antioxidant defenses—and the decrease in Keap1 protein levels, which normally suppresses Nrf2 activity (Wang et al., 2022; Liu and Guo, 2024). Another tannin source, *in vitro* digested chestnut and quebracho, were also shown to increase cell viability in a model of oxidative stress induced with hydrogen peroxide and dextran sodium sulfate further (Reggi et al., 2020). These findings underscore the potential of tannins as a protective agent against oxidative damage, especially relevant in conditions like PWD.

Oxidative and inflammatory processes are closely interrelated, and TA’s dual capacity to mitigate both stresses in IPEC-J2 model is well documented. TA treatment was shown to reduce the levels of pro-inflammatory markers, such as TNF-α, IL-1β and IL-6, as well as the protein abundance of TLR4, MyD88, phosphorylated NF-κB, and NLRP3, in cells exposed to TBH- or ETEC-induced inflammation (Wang et al., 2022; Liu and Guo, 2024). These results indicate that TA may reduce inflammatory responses by modulation of components of the TLR4-MyD88-NF-κB-NLRP3 pathway, which are known to mediate inflammatory responses (Liu and Guo, 2024). TA was also shown to modify the protein levels of LC3 and SQSTM1/p62 in TBH-treated cells, indicating that it may regulate autophagy, a cellular process responsible for removing damaged organelles, proteins, and pathogens, to maintain cellular health and prevent further inflammation (Wang et al., 2022).

Most of the studies on the anthelmintic properties of tannins have been performed *in vivo*, but some important *in vitro* studies have also been reported. Diverse tannin-rich plant extracts and isolated CT have shown strong *in vitro* anthelmintic activity against *Ascaris suum*, affecting larval migration, survival, and tissue integrity (Williams et al., 2014a) as well as against *Oesophagostomum dentatum*, affecting especially larval development and motility of adult worms (Williams et al., 2014b). These findings highlight the potential of tannins as effective anthelmintic agents in pigs, offering a promising strategy to reduce reliance on conventional synthetic drugs.

## 4 Health benefits of tannins from *in vivo* studies

### 4.1 Impact on pig production

#### 4.1.1 Influence on growth performance

Tannins have been studied for their impact on growth performance, with several reviews highlighting both their potential benefits and limitations. For example, a meta-analysis focused on weaned piglets showed that dietary tannins, especially from chestnut and grape seeds, have positive effects on animal performance and physiological traits (Nuamah et al., 2024). Similarly, a review on the inclusion of grape by-products in pig and poultry diets demonstrated that supplementation with grape by-products up to 9% positively influences performance and weight gain in pigs (Costa et al., 2022). A separate review focussed on chestnut and quebracho tannins emphasising their effects on performance and intestinal health during the post-weaning period and the later life of fattening pigs (Caprarulo et al., 2021). From a broader perspective, a review of the chemical properties and biological activities of tannins across animal species underscored their antimicrobial, anti-inflammatory, and antiparasitic potential, but also anti-nutritional effects and inconsistent efficacy in monogastric species (Huang et al., 2018). These reviews collectively support the potential of tannins as growth-supporting feed additives, though they also underline the need for further characterization regarding their mechanisms of action and optimal application in pig production systems.

Studies not covered in the aforementioned reviews are summarized in Supplementary Table S1. Several of these reported neutral effects on performance across various tannin sources, including, for example quebracho, gallnut, and black wattle, as well as across different inclusion levels (Deng et al., 2024; Ma et al., 2024; Schneider et al., 2024). A 1% inclusion of a coca bean extract in an infection trial resulted in the same feed conversion ratio (FCR) and average daily feed intake (ADFI) but lower average daily gain (ADG) after infection, compared to the control (Verhelst et al., 2014). In contrast, a natural tannin supplement (2,000 mg/kg) improved ADG in ETEC-infected piglets on days 8 and 9 post-infection compared to infected piglets without tannin supplement, despite lower feed intake compared to controls (Zhang et al., 2023).

In another study, a diet with 2,000 mg/kg condensed tannins (black wattle extract) in 22-day old weaned piglets over a period of 42 days tended to increase ADG over the whole trial period, while the ADFI, ADG, body weight (BW) and gain-to-feed ratio (G:F) were significantly better than in the control group during days 29–43 (Souza et al., 2025). Chestnut tannins that were coated with hydrogenated palm oil resulted in a higher ADG and lower feed-to-gain ratio (F/G) ratio (Xu et al., 2022). A diet with 20% carob pulp (partly supplemented with vitamin E) from 130 to 169 days of life, improved FCR between days 130 and 151 by 4% in comparison to the control group, but no difference was observed over the total trial period (Bottegal et al., 2024). Taken together, condensed tannins have been researched most intensively and can have positive effects on the performance of the animals, though many studies report neutral effects.

Evidence from existing studies indicates that the effects of tannins on growth performance can vary depending on the type of tannin, as well as the dosage and duration of supplementation. Tannins differ fundamentally in their chemical structure, molecular weight, and stability, which directly influence their bioactivity. HT, composed of gallic or ellagic acid units esterified to a core sugar, are more readily hydrolysed in the gastrointestinal tract, releasing bioactive phenolics that exert antioxidant and antimicrobial effects. CT (proanthocyanidins) are polymers of flavan-3-ols, more resistant to degradation, and primarily exert their effects within the gut lumen by modulating microbial populations and gut morphology. These structural differences may explain variations in growth performance outcomes. HT, due to their higher reactivity and bioavailability, can have more pronounced antioxidant and anti-inflammatory effects, supporting gut barrier integrity and reducing weaning-associated stress. CT, while less bioavailable, may contribute more effectively to pathogen inhibition in the gut through protein precipitation and microbiota modulation (Liu H. et al., 2020; Souza et al., 2025).

It is not fully understood how tannins may affect the piglet performance (Caprarulo et al., 2021; Nuamah et al., 2024) and further research is warranted. Improvements in FCR may be linked to several factors, including enhanced intestinal metabolism, protection of intestinal morphology, and a reduction in intestinal diseases—all of which can influence piglet’s performance (Nuamah et al., 2024). Based on a meta-analysis of 16 studies, a more differentiated understanding of the effects of various tannin sources on piglet performance has been provided (Nuamah et al., 2024).

#### 4.1.2 Influence on apparent nutrient digestibility

Numerous studies have investigated the effects of tannins on nutrient digestibility in pigs, with varied outcomes depending on tannin type, source, and inclusion rate (Supplementary Table S1). Overall, tannins, particularly at higher inclusion levels, tend to negatively impact the apparent ileal digestibility (AID) and apparent total tract digestibility (ATTD) of crude protein (CP) and energy. In weaner pigs, 100 mg/kg of grape seed extract reduced the ATTD of CP after 14 days, while 150 mg/kg led to a significantly lower CP ATTD than all other groups. After 28 days, CP ATTD remained lowest in the 150 mg/kg group. The AID of CP was significantly reduced by 100 and 150 mg/kg at day 14, but only the 150 mg/kg group showed a reduction at day 28. High tannin levels also impaired gross energy digestibility. At day 14, the 150 mg/kg group showed lower AID and ATTD of gross energy compared to control and 50 mg/kg. After 28 days, only AID remained reduced. Additionally, AID of dry matter (DM) and ether extract was lower in the 100 and 150 mg/kg groups after 28 days (Li et al., 2020).

Similarly, a linear decline in the ATTD of DM, CP, gross energy, and crude fibre was observed with increasing dietary inclusion of TA from gallnut. However, the ATTD of ether extract was improved at the highest inclusion level (0.4%) (Song et al., 2021). Grape tannins with an inclusion of 1.5% reduced threonine and isoleucine AID (Myrie et al., 2008). When feeding 4% quebracho extract, it was observed that the composition of the endogenous amino acids changed and the quantity of endogenous nitrogen was elevated, in particular, proline, arginine, glycine and leucine. In contrast, threonine, glutamic acid, serine and isoleucine were less abundant. The apparent nitrogen digestibility of the diet with quebracho extract was significantly lower than of the control. In addition, the true nitrogen digestibility (measured with the peptide alimentation ultrafiltration method) was decreased in the tannin group. However, with the ^15^N-isotpe dilution method no difference in the real nitrogen digestibility was observed (Steendam et al., 2004). The inclusion of condensed tannins from black wattle (2,000 mg/kg) did not affect DM, CP, crude ash and gross energy ATTD (Souza et al., 2025). An extract from dried grapes (150 mg/kg) in diets for weaned pigs resulted in an improvement of the ATTD of DM, organic matter, gross energy and acid-hydrolysed ether extract as well as CP and P in relation to the control (Rajković et al., 2021). Coated chestnut tannins increased the ATTD of CP. No impact was observed on crude fat, crude ash, Ca and P (Xu et al., 2022). It has been shown in these studies that tannins have different effects on the digestibility of nutrients and in particular on CP. Higher dietary inclusion rates can have a more negative impact. Notably, some studies observed no significant impact of tannins on digestibility. However, coated tannins can also have a positive effect on the ATTD of CP (Xu et al., 2022).

In a trial where low or high tannin sorghum were fed to pigs, the high sorghum variant resulted in a reduction of AID and ATTD of DM, gross energy and CP. Especially lysine, threonine, valine, histidine, arginine, serine, glutamic acid and aspartic acid exhibited a reduced AID (Pan et al., 2022a). In another trial with four high-tannin sorghum varieties and four low-tannin sorghum varieties, it was also observed that the high sorghum varieties had a lower AID, ATTD and hindgut digestibility of gross energy and CP. In addition, the standardised ileal digestibility of the above-mentioned amino acids was also lower in the high tannin sorghum diet (Pan et al., 2022b).

In the diets of finishing pigs, tannins negatively affected digestibility only at higher inclusion levels. Inclusion of 0.5% chestnut wood extract resulted in a lower ATTD of DM and nitrogen, while 0.25% did not have an impact, however, diets did not have the same crude protein content (Antongiovanni et al., 2007). A study comparing four sorghum varieties found that the diet with the lowest tannin content (1.4 g/kg DM) had the highest AID of DM and amino acids. The medium-tannin group (4.6 g/kg DM) showed the lowest CP and amino acid AID, while the higher-tannin groups (9.8 and 10 g/kg DM) showed intermediate or mixed results. Proline AID decreased with increasing tannin levels (Mariscal-Landín et al., 2004).

Inclusion level of 5.3 g/kg chestnut tannin extract did not result in different ATTD of DM, organic matter, ether extract and CP. Moreover, no impact of tannin addition was observed on the urine and faecal N excretion, when the two groups with the same CP content in the diet were compared (Galassi et al., 2019). A supplementation of diets with 0, 5, 10 or 15% chestnut meal led to reduced ATTD of DM, EE, crude ash, tannin and CP with higher inclusion levels in the diet (Lee et al., 2016). Lower ATTD of CP was observed when pigs received a diet with 20% carob pulp, while the ATTD of EE and hemicellulose was increased (Bottegal et al., 2024). In growing and finishing pigs, it has been shown that adverse effects, particularly with regard to the digestibility of DM and CP, mostly occur when higher quantities of tannins or tannin-containing feedstuff are used. Moreover, some amino acids seemed to be more sensitive to tannins than others. In contrast, tannin addition might not have a great impact on ether extract digestibility.

It is known that especially proteins with a large size, an open loose structure as well as with a higher content of hydrophobic amino acids and proline reveal a higher affinity to tannins (Mehansho et al., 1987; Cappai et al., 2013). In contrast proteins with a smaller, dense structure and disulfide bonds show the lowest affinity of proteins to react with tannins. Prolines higher affinity to bind tannins relies likely on the fact that it do not fit into the α-helix. Due to that, the occurring structure is more open and not so dense which makes it more accessible to the tannins and hydrophobic bond can be built up more simple (Mehansho et al., 1987). Another influence factor is the pH as the bondage between the condensed tannin and the protein is stronger when the pH is around the isoelectric pH of the protein (Girard and Bee, 2020).

In summary, the impact of tannins on nutrient digestibility in pigs is highly variable and influenced by factors such as tannin type, source, and inclusion level; while higher inclusion rates often impair the digestibility, some tannin sources or forms—particularly when used at lower levels—can have neutral or even beneficial effects, highlighting their potential role in pig nutrition.

#### 4.1.3 Influence on incidence of diarrhea

As mentioned previously, PWD is a common and economically important issue in pig production, often associated with gut colonization by ETEC during periods of stress. Tannins have gained attention as a potential non-antibiotic and non-ZnO strategy to mitigate this condition. Challenge trials involving ETEC infection in piglets fed tannins were reviewed previously (Girard and Bee, 2020; Canibe et al., 2022). One of these reviews focused on the preventive use of dietary tannins, highlighting their potential to protect piglets against coliform infections and describing their effects on pathogenic bacteria based on both *in vitro* and *in vivo* studies. The authors concluded that tannins may reduce the incidence and severity of PWD (Girard and Bee, 2020). The other review provided a broader overview of strategies to prevent the incidence of PWD and suggested that the inconsistent results observed across studies may be due to differences in tannin sources, structures, and inclusion levels (Canibe et al., 2022).

Since the publication of those reviews, two more ETEC-challenge trials were conducted (Supplementary Table S1). In the first, supplementation with condensed tannins (1 g/kg) led to reduction in diarrhea rate and index of diarrhea in comparison to the control group (Yi et al., 2023). In the second, supplementation with 7.5 g/kg quebracho and chestnut extract did not lead to a difference in average duration of diarrhea, average daily prevalence of diarrhea or ETEC F4 excretion, whereas the tannin group showed a better fecal score than the control group (Ollagnier et al., 2025).

Other trials, although not conducted under ETEC challenge and therefore not included in the aforementioned reviews, still provide supportive evidence for the anti-diarrhea effects of tannins (Supplementary Table S1). In two trials with tannins, no diarrhea was observed. Whereby, one trial was carried out with 23 days-old piglets and 0.35% chestnut extract and 0.16% organic acids in the diet (Brus et al., 2013b). In the other study, quebracho extract was used in two different dosages (0.5% and 1.0%) in 21 days-old piglets. Only the piglets fed with the higher dosage developed some scours. However, it did not spread between the animals and otherwise solid faeces were observed (Ma et al., 2024). When the same product was added at a concentration of 0.3% in a diet for piglets of the same age, no diarrhea was recorded. Whereas 12.5% of diarrhea incidence was detected in the control and the group with 0.2% of this product (Ma et al., 2021). An addition of 2.5 g/kg black wattle extract to diets led to a reduction of pasty and liquid faeces during the first days after weaning (d 0–7) and a decrease of pasty faeces thereafter (d 8–22), while only a tendency was recorded for lower incidence of liquid faeces during that period (Schneider et al., 2024). Likewise, a supplementation of 2,000 mg/kg condensed tannins from black wattle resulted in a reduction of diarrhea (Souza et al., 2025). A linear reduction in diarrhea rate and severity occurred with increasing inclusion levels (0.05%–0.4%) of TA from gallnut (Song et al., 2021). These studies suggest that tannins may have protective effects, however, both tannin type and dosage clearly influence the outcomes.

The effectiveness of tannins may be related to their bacteriostatic properties, such as their ability to inhibit bacterial growth, adherence to the intestinal epithelium, and biofilm formation in the gastrointestinal tract of piglets. Moreover, tannins can hinder the production and activity of enterotoxins. These possible mechanisms have been described in more detail in a previous review (Girard and Bee, 2020).

### 4.2 Modulation of the intestinal microbiome

The gastrointestinal microbiota plays a critical role in pig health, particularly during stressful periods such as weaning. Beyond their antimicrobial effects, tannins have been shown to act as eubiotics influencing the structure and composition of the gut microbiota, potentially promoting a more balanced microbial ecosystem (Huang et al., 2018; Caprarulo et al., 2021). In the following, only recent studies that are not listed in the previous reviews are highlighted (Table 3).

**TABLE 3:** Impact of tannins on microbiota composition and metabolic activity.

Source	Feed supplement (form)	Tannin content	Type of tannin^a^	Tested model	Changes in microbiota composition and metabolic activity^b^	Dose of feed supplement	Duration	References
Chestnut (*Castanea sativa* Mill.)	Coated tannins (commercial product, hydrogenated palm oil was used as coating material)	25%	HT	180 weaned barrows (Duroc x Landrace x Yorkshire), 28 days-old, average BW: 8.6 kg	Chao 1, Observed species, Shannon and Simpson indices = Lachnospiraceae was the most abundant family in the Con group, while Ruminococcaceae was the most abundant family in the Tan group ↑ rel. abundance of *Faecalitalea*, *Acidaminococcus*, *Methanobrevibacter smithii*, *Faecalibacterium* and *Turicibacter* ↑	Con: basal diet (include ZnSO_4_) Tan: basal diet + 1,500 mg/kg coated tannins Add. ZnO: basal diet + ZnO (Zn content 1,600 mg/kg)	20 days	Xu et al. (2022)
Chestnut wood extract (*Castanea sativa* Mill.)	Farmatan (commercial product)	75% tannins	HT	48 weaned piglets (German Landrace x Pietrain), 28 days-old, average BW: 8.23 ± 0.93 kg housed in metabolic cages 4 days adaption period (all same diet)	number of viable lactobacilli ↑ (tended) in jejunum digesta number of coliforms ↑ (tended) in caecum digesta Ammonia concentration ↓ (tended) in caecum digesta propionic acid ↓ (tended) in caecum digesta in 1.13 and 4.5 g/kg group in relation to 2.25 g/kg and Con iso-butyric and iso-valeric acid ↓ in caecum digesta *n*-butyric acid and total volatile fatty acids ↓ in 1.13 g/kg and 4.5 g/kg than in the others	Con: basal diet 1.13 g/kg: basal diet + 1,130 mg/kg tannins 2.25 g/kg: basal diet + 2,250 mg/kg tannins 4.5 g/kg: basal diet + 4,500 mg/kg tannins Corn was replaced by the tannin product	28 days	Biagi et al. (2010)
Quebracho tree tannin extract and Chestnut tree tannin extract (*Schinopsis* spp., *Castanea* spp.)	Silvafeed Nutri P/ENC for Swine (commercial product)	75 g tannins/100 g DM	CT and HT	120 weaned piglets (Large White x Landrace), 28-day-old piglets	Principle coordinate analysis did not result in a different microbiota of the piglets feces, while PERMANOVA show a separation between the groups in terms of the microbiome Chao 1 = in feces Observed species, Shannon and Simpson indices ↑ in feces Rel. abundance of *Bacteroidetes* and *Actinobacteria* ↓, *Spirochaetae* and *Cyanobacteria* in feces ↑ Rel. abundance of Clostridiaceae as well as Peptococcaceae Spirochaetaceae as well as Peptostreptococcaceae ↑, rel. abundance of Prevotellaceae, Eubacteriaceae*,* Coriobacteriaceae*,* Desulfovibrionaceae*,* Veillonellaceae*,* Rikenellaceae as well as Deferribacteraceae*,* ↓ Rel. abundance of *Shuttleworthia*, *Pseudobutyrivibro*, *Anaerostipes*, *Solobacterium* and *Peptococcus* ↑, rel. abundance of *Synotrophococcus*, *Mitsuokella*, *Sharpea*, *Atopbium* and *Prevotella* ↓ Butyrate in feces ↑, valerate in feces ↓	Con: basal diet Ch/Qu: basal diet + 1.25% extract (quebracho tree tannin extract and chestnut tree tannin extract)	One week of adaptation (all basal diet) + 40 days treatment diets	Miragoli et al. (2021)
Gallnut (*Galla chinensis)*	Tannic acid (TA) (commercial product)	—	HT	432 weaned piglets, average BW: 7.05 ± 1.05 kg	Rel. abundance of *Proteobacteria* were higher and *Firmicutes* in 1.5 g/kg TA ↓ in cecal digesta compared to Con Shannon index = Operational Taxonomic Units ↓ due to the inclusion of 3 g/kg TA Rel abundance of *[Eubacterium] oxidoreducens group* and *Escherichia-Shigella* ↓ in 3.0 g/kg TA than in Con, relative abundance of *Candidatus Brocadia* ↓ in 3.0 g/kg TA and 1.5 g/kg TA in comparison to the Con	Con: basal diet 1.5 g/kg TA: basal diet + 1,500 mg/kg TA 3.0 g/kg TA: basal diet + 3,000 mg/kg TA 1.8 g/kg ZnO: basal diet + 1,800 mg/kg ZnO	21 days	Deng et al. (2024)
Gallnut (*Galla chinensis)*	Tannalbin (commercial product)	51% tannic acid and 40.17% protein	HT	180 weaned piglets (Duroc x Landrace x Yorkshire) 21 days-old; average BW: 7.77 ± 0.17 kg 5 days adaptation period	*Bacillus* number in caecum digesta ↑ TA (dose dependent), *Bacillus* counts ↑ in colon digesta of 0.4% TA in relation to Con, 0.1% and 0.2% TA *E. coli*. in colon digesta ↓ of 0.1% TA piglets in comparison to Con and 0.4% TA as well as reduced value ↓ in 0.4% TA in relation to 0.05% TA Cecum digesta: Butyric acid ↑ in all TA groups, isovaleric acid ↑ in 0.1%, 0.2% and 0.4% group than in Con, propionic acid ↑ in 0.2% TA than in 0.1, 0.05% TA and Con, and 0.4% TA ↑ than the Con Colon digesta: acetic acid ↑ in 0.4% TA than in the others, isobutyric acid ↑ in 0.05% TA in relation to 0.2% and 0.4% TA and 0.4% TA ↓ than 0.1% TA	Con: basal diet 0.05% TA: basal diet + 0.1% tannalbin 0.1% TA: basal diet + 0.2% tannalbin 0.2% TA: basal diet + 0.4% tannalbin 0.4% TA: basal diet + 0.8% tannalbin	29 days	Song et al. (2021)
Black wattle (*Acacia mearnsii*)	Tanfeed (commercial product)	Approximately 47.8% tannins,	CT	200 weaned piglets (PIC), 22 days-old piglets, average BW: 6.0 ± 0.9 kg	Chao1 and Shannon indices in rectum content = Rel. abundance of *Brevibacillus* spp. in rectum ↑, *Enterococcus* spp. abundance in rectum =	Con: basal diet ENR + ZnO: basal diet + 10 mg/kg of enramycin + 2,500 mg/kg of zinc oxide during 21 days BUT: basal diet + 900 mg/kg of sodium butyrate TAN: basal diet + 2,000 mg/kg of condensed tannin	42 days 4 feeding phases	Souza et al. (2025)
Extract of dried grapes (*Vitis vinifera*)	Water-based grape extract (commercial product)	>40% total polyphenols, > 30% procyanidins, < 5% water	CT	180 weaned piglets (DanBred x Piétrain) 23 days-old piglets; average BW: 6.9 ± 0.1 kg	Microbial metabolites in ileum digesta = , Lactic acid in colon digesta ↑ (tendency)after 27/28 days, ammonia content in ileum digesta ↓ (tendency) in relation to Con after 27/28 days	Con: basal diet PC: basal diet + amoxicillin for the first 5 days of the trial GE: basal diet + 150 mg/kg grape extract	56 days starter period: 1–13 days grower period: 14–56	Rajković et al. (2021)
Kenwood	—	—	CT	72 weaned piglets (Duroc x Landrace x Yorkshire), 26 days-old, average BW: 8.40 kg ETEC-K88- challenged environment; bacterial solution (6 × 10^8^ CFU/mL) was sprayed on day 1, 5, 9, 13, 17, 21, and 25 of the trial	BW, ADG, ADFI and F/G = Reducing rate and index of diarrhea ↑ (d 0–14, 15–28 and 0–28) Chao1, Shannon and Simpson = in colon digesta Microbial composition = in colon digesta	Con: basal diet ZnO: basal diet + 1.5 g/kg zinc oxide CT: basal diet + 1 g/kg condensed tannins ZnO + CT: basal diet + 1.5 g/kg ZnO + 1 g/kg condensed tannins	28 days	Yi et al. (2023)

^a^ HT, hydrolysable tannins; CT, condensed tannins.

^b^ = no change; ↑, increase; ↓, decrease.

Supplementation of 1.5 g/kg TA from gallnut to a diet for weaned piglets led to a higher abundance of *Proteobacteria* and a decrease of *Firmicutes* in the caecum digesta compared to piglets of the control group. This effect was not observed in a group with 3 g/kg supplementation. In contrast to that, the group with 3 g/kg TA supplementation had a lower abundance of *Eubacterium oxidoreducens* and *Escherichia-Shigella* compared to control animals. *Candidatus brocadia* was reduced in the 1.5 and 3 g/kg TA supplementation. The authors hypothesized that the altered composition of bacteria in the 3 g/kg group might be related to the higher level of antioxidants in the gut due to the TA supplementation (Deng et al., 2024). Supplementation of 0.05, 0.1, 0.2% and 0.4% TA from gallnut resulted in linear increase of *Bacillus* spp. in the caecum content. *Escherichia coli* numbers in colon content were lower in 0.1% TA group than in 0% and 0.4% TA. Moreover, 0.05% and 0.2% TA addition resulted in higher counts of total bacteria in caecum content than in 0.4% group (Song et al., 2021). An addition of 2,000 mg/kg tannins of black wattle tannins led to a higher abundance of *Brevibacillus* spp. in rectum content than in control piglets (Souza et al., 2025). A diet with 1.25 chestnut/quebracho extract decreased the abundance of *Bacteroidetes* and *Actinobacteria* while *Spirochaetae* and *Cyanobacteria* increased. Moreover, Clostridiaceae, Peptococcaceae, Spirochaetaceae and Peptostreptococcaceae had a higher abundance and *Prevotellacea,* Eubacteriaceae*,* Coriobacteriaceae*,* Desulfovibrionaceae*,* Veillonellaceae*,* Rikenellaceae*, and* Deferribacteraceae a lower abundance in the tannin group. On the genus level, the rel. abundance of *Shuttleworthia*, *Pseudobutyrivibro*, *Anaerostipes*, *Solobacterium* and *Peptococcus* was higher and *Synotrophococcus*, *Mitsuokella*, *Sharpea*, *Atopbium* and *Prevotella* were lower in the feces of tannin supplemented piglets (Miragoli et al., 2021). When coated chestnut tannins were supplemented to the diet of weaned pigs, Ruminococcaceae dominated followed by Lachnospiraceae where it was the other way around for the control. The addition of coated tannins resulted in higher abundance of the genera *Faecalibacterium, Faecalitalea*, *Acidaminococcus*, *Methanobrevibacter smithii*, and *Turicibacter* than in the control group in the digesta of the same segment as before (Xu et al., 2022).

Mentioned studies also examined the impact of tannin supplementation on microbial diversity and richness, revealing inconsistent changes in Shannon, Simpson, and Chao1 indices. Shannon index in caecum digesta did not differ due to TA supplementation (Deng et al., 2024) nor were Shannon and Simpson indices different in colon digesta after addition of coated tannins (Xu et al., 2022). In addition, Chao 1, Shannon and Simpson in colon digesta were unaffected by supplementation of condensed tannins in a trial with a challenged environment (Yi et al., 2023). Also, an addition of black wattle tannins had no impact on the Shannon index in rectum digesta (Souza et al., 2025). Moreover, the Chao 1 was unaffected in the later study (Souza et al., 2025) and in faeces samples from a trial with 1.25 chestnut/quebracho extract (Miragoli et al., 2021) as well as in the colon digesta of piglets that received an addition of coated tannins in the diet (Xu et al., 2022). In contrast to that 3 g/kg TA led to a reduction in OTUs in caecum content related to the control (Deng et al., 2024), while an addition of coated tannins to a diet had no impact on observed species in colon digesta (Xu et al., 2022). Contrary, in a trial with 1.25% chestnut/quebracho extract the observed species, Shannon and Simpson indices were increased (Miragoli et al., 2021).

The studies show that tannin supplementation can have an impact on the bacterial composition and may lead to a shift on phyla and some families or genera, while the observations are very diverse. This might be also related to the different dosages and tannin sources. The stability of diversity indices, despite compositional shifts, suggests that tannins reorganize the gut microbiota without reducing richness. This reorganization may favour beneficial taxa, such as short-chain fatty acids (SCFA) producers, which support gut health through improved barrier function and anti-inflammatory effects.

Variability across studies is likely driven by tannin type, source, concentration, and form of administration, all of which influence bioactivity. To better understand functional outcomes, future research should combine microbiota profiling with metabolomic or metagenomic analyses. This integrated approach will clarify how tannins modulate microbiota function and contribute to gut health in piglets.

### 4.3 Effects of tannins on microbial metabolites

The metabolic pathways by which tannins are degraded by the microbiome, along with information on their stability and the absorption of tannin compounds, were described in a previous review (Girard and Bee, 2020). Tannins can also affect microbial metabolites in the gastrointestinal tract. However, only a few *in vivo* studies have addressed the change of metabolite composition within the digesta (Table 3).

Dietary inclusion of TA from gallnut (0.05%–0.4%) altered the SCFA profile in the cecal and colonic digesta. Specifically, 0.2% TA resulted in the highest concentrations of acetic and propionic acids in the caecum, while all tannin-supplemented groups had elevated levels of butyric acid. A dose-dependent increase in propionic, butyric, and valeric acids was observed in the caecum, while acetic acid was elevated in the colon at the highest inclusion level. Conversely, isobutyric acid decreased linearly with increasing tannin dosage (Song et al., 2021). Faecal butyrate concentration increased when 1.25% chestnut/quebracho extract was included in the diet, while valerate concentration decreased compared to the control group (Miragoli et al., 2021). Moreover, the animals fed diets with tannins showed an increased concentration of phenolic compounds and nitrogen, whereas ammonia and urea concentrations were not altered (Caprarulo et al., 2020).

A supplementation of a weaner diet with 150 mg/kg of grape extract resulted in a tendency towards a higher lactic acid content in colon digesta although the effect diminished at later stages of the experiment. The supplementation had no effect on the short chain fatty acids and lactate concentrations in the ileum digesta. Ammonia and composition and biogenic amines in colon content were unaffected by the addition of tannins to the diet. In contrast to that a decrease in ammonia concentration in ileum digesta was observed after 27/28 days of supplementation in comparison to piglets fed the control diet (Rajković et al., 2021). In addition, lower iso-butyric and iso valeric acid concentrations and tended lower ammonia concentrations in caecum digesta were observed in a trial, where 1.13 g/kg, 2.25 g/kg, and 4.5 g/kg were added to the basal diet (Biagi et al., 2010). In summary, diverse effects on postbiotic concentrations could result from different tannin sources and tannin dosages.

### 4.4 Impact of tannins on intestinal morphology

The main site of digestion and absorption of macro- and micronutrients is the small intestine, which consists of duodenum, jejunum and ileum. The small intestine has villi and microvilli, which increase the surface area for absorption. Longer, more developed villi contribute to a larger villous surface area. This in turn enhances nutrient absorption, promotes growth, feed conversion efficiency, and overall health and nutrition in pigs (Itza-Ortiz et al., 2019). At the base of the villi are crypts, where new epithelial cells form and migrate upward to replace old cells. Smaller crypts and faster epithelial cell turnover are associated with better gut health and nutrient absorption efficiency, while deeper crypts may indicate intestinal damage or inflammation, which can reduce the pig’s ability to properly digest and absorb nutrients (Spreeuwenberg et al., 2001).

Weaning triggers distinct morphological and histological changes in the piglet’s small intestine, including a reduction in villous height (VH) and villous surface area, as well as an increase in crypt depth (CD) (Spreeuwenberg et al., 2001). Recent studies have highlighted the beneficial effects of tannin supplementation on gut morphology (Table 4).

**TABLE 4:** Impact of tannins on intestinal morphology and epithelial health.

Source	Feed supplement (Form)	Tannin content	Type of tannin^a^	Tested model	Changes in gut microarchitecture^b^	Dose of feed supplement	Duration	References
Chestnut (*Castanea sativa* Mill.)	The hydrolysable tannins product (commercial product)	≥75% tannins	HT	Weaned piglets [(Landrace × Yorkshire) × Duroc, 28 days-old, average weight of 7.81 ± 0.99 kg]	VH/CD ratio in ileum ↑, VH in jejunum ↑, VH in ileum tended to ↑	1,000 mg/kg	28 days	Liu et al. (2020a)
Chestnut (*Castanea sativa* Mill.)	Farmatan, (commercial product)	75% tannins, mainly gallotannins	HT	Crossbred piglets (German Landrace 6 Pietrain) weaned at 28 days old	Ileal CD tended to ↓	2,250 mg/kg and 4,500 mg/kg	28 days	Biagi et al. (2010)
Chestnut (*Castanea sativa* Mill.)	Farmatan (commercial product)	75% tannins, mainly gallotannins	HT	fattening boars (crosses of Landrace × Large white) of 52 kg	Apoptotic cell count ↓, the most notable effect in the ascending colon (16%–32%)	1%, 2% and 3% of extract	70 days, 52–122 ± 10 kg	Bilić-Šobot et al. (2016)
					Mucosal thickness ↑ in duodenum, mitotic cell counts ↓ in caecum 46%, in the descending colon −46%, apoptotic cell count ↓ significant for caecum and a tendency for descending colon	Group no. 1 – 1%		
					Mitotic cell counts ↓ in caecum - 64%, in the descending colon - 62%	Group no. 2 – 2%		
					VH ↑ (23%), and villus surface area ↑ (35%–38%), the ratio of VH/CD tended to improve ↑ in duodenum; mitotic cell counts ↓ in caecum 71%, in the descending colon - 69%	Group no. 3 – 3%		
Chestnut (*Castanea sativa* Mill.)	Farmatan (commercial product)	75% tannins, main	HT	fattening pigs (from 30 kg to 120 kg body weight)	Narrower crypt/villi ratio in the small intestine	2,000 mg/kg (0.2%)	fattening period from 30 to 120 kg	Brus et al. (2013a)
Oak tannin *(Quercus* spp.*)*	CONTAN (commercial product)	-	HT	fattening pigs (from 30 kg to 120 kg body weight)	VH ↑ Narrower crypt/villi ratio in the small intestine	2,000 mg/kg (0.2%)	fattening period from 30 to 120 kg	Brus et al. (2013a)
Gallnut (*Galla chinensis*)	TA (commercial product)	TA	HT	weanling pigs (7.05 ± 1.05 kg)	VH ↑ in duodenum and jejunum by 7.7%–25.0% ↑, the ratio VH/CD ↑ in the duodenum and jejunum, CD ↓ in jejunum and ileum by 10.1%–11.5% ↓	3,000 mg/kg	21 days	Deng et al. (2024)
Gallnut (*Galla chinensis*)	Tannalbin (commercial product)	51% TA	HT	Crossbred piglets (Duroc × Landrace × Yorkshire), weaned at 21 ± 1 d of age with an initial average body weight of 6.60 ± 0.27 kg,	CD ↓, VH/CD ratio ↑ in the duodenum	1,973.09 mg/kg (0.2%)	28 days	Yu et al. (2020)
					Tended to reduce CD ↓, VH ↓ of the ileum	12,004.84 mg/kg (1%)		
Gallnut (*Galla chinensis*)	Tannalbin (commercial product)	51% TA	HT	weaned piglets (Duroc × Landrace × Yorkshire, 24 days of age, initial average BW ¼ 7.77 ± 0.17 kg)	VH in jejunum ↑	0.05%, 0.2% and 0.4% TA	28 days	Song et al. (2021)
					VH in duodenum ↑	0.4% TA		
Chinese gallnut (*Galla chinensis*)	Chinese gallnut (commercial product)	TA	HT	Duroc × [Landrace × Yorkshire] (initial body weight = 5.99 ± 0.13 kg, weaned days = 21 days)	A lower duodenal CD ↓ A higher ratio VH/CD↑ in the duodenum	1,000 mg/kg	14 days	Wang et al. (2020)
Chinese gallnut (*Galla chinensis)*	Hydrolytic Chinese gallnut TA (GCT) (commercial product)	The effective content of TA 70.38%	HT	weaned piglets (average initial weight 10 ± 0.2 kg) of 31 days old	CD in ileum ↓ A control group, with a basal diet including + 1,600 mg/kg ZnO; ZnO and premix were mixed into diet.	1,899.5 mg/kg GCT (0.2%)	21 days	Sun et al. (2021)
Quebracho (*Schinopsis* spp.)	Quebracho tannin product (MGM-P) (commercial product)	CT - more than 50% of the overall extract	CT	Piglets (Duroc × Landrace × Yorkshire), at 21 days of age	VH in jejunum ↑, CD in ileum ↓, thinner colonic mucosae ↓	3,000 mg/kg (0.3%)	20 days	Ma et al. (2021)
*Vitis vinifera*	Dried extract from dried grapes (commercial product)	Total polyphenols > 40%	CT	Weaning piglets (6.9 ± 0.1 kg), (DanBred × Piétrain), at weaning (23 ± 1 day of age)	VH ↑, villus surface area ↑in jejunum on day 27/28; VH ↑ and villus surface area ↑ in ileum on day 55/56, VC ratio ↑ and number of goblet cells ↑;	150 mg/kg	8 weeks	Rajković et al. (2022)
Grape seed (*Vitis* spp.)	Grape seed proanthocyanidins (GSPs) (commercial product)	—	CT	Crossbred weaned piglets (Duroc × Landrace × Large White, n = 120, weaned at 28 days)	VH ↑, CD ↓ in jejunum and ileum, VH/CD ratio in jejunum and ileum ↑	250 mg/kg GSPs	28 days	Han et al. (2016)
-	Natural-based tannin (NBT) (commercial product)	—	—	7-day-old healthy crossbred piglets (Duroc × Landrace × Yorkshire) challenged with ETEC	VH ↑ and surface area ↑ in duodenum and ileum; VH/CD ratios ↑ in duodenum, jejunum and ileum; CD ↓ in all intestinal sections compared to the ETEC group	2,000 mg/kg	9 days	Zhang et al. (2023)

^a^ HT, hydrolysable tannins; CT, condensed tannins.

^b^ ↑, increase; ↓, decrease.

It was shown that tannins consumption enhances VH in intestines. In the duodenum, higher VH was observed after supplementation with HT from chestnut (Bilić-Šobot et al., 2016) and gallnut (Song et al., 2021; Deng et al., 2024). In the jejunum, supplementation with tannins from chestnut, quebracho, and grape has been shown to enhance VH (Han et al., 2016; Liu H. et al., 2020; Ma et al., 2021). Similar positive effects have been reported for gallnut and grape tannins by other authors (Song et al., 2021; Rajković et al., 2022; Deng et al., 2024). Furthermore, HT from chestnut and CT from grapes positively affected ileal VH (Han et al., 2016; Liu H. et al., 2020; Rajković et al., 2022). However, TA at a concentration of 1% was reported to negatively affect VH in the ileum suggesting that the positive effect of tannins may be dose-dependent (Yu et al., 2020).

By enhancing VH, tannin supplementation was shown to positively affect villous surface area. An increase in surface area in duodenum was observed in piglets supplemented with 3% chestnut wood extract (Bilić-Šobot et al., 2016). In the jejunum and ileum, greater villus surface area was observed following the supplementation with dried grape extract (Rajković et al., 2022). Moreover, tannin supplementation in the diet of piglets challenged with ETEC significantly increased VH and, consequently, the surface area in both the duodenum and ileum (Zhang et al., 2023).

Supplementation with tannins was also shown to decrease CD, minimizing the stress-induced changes. TA reduced CD in the duodenum (Wang et al., 2020; Yu et al., 2020). Similarly, reductions in CD within the jejunum and ileum were observed following the application of TA (Deng et al., 2024) and CT from grapes (Han et al., 2016). A tendency for reduction of ileal CD was also observed after supplementation with HT from chestnut (Biagi et al., 2010) and TA from gallnut (Yu et al., 2020). In another study, a decrease in ileal CD was noted with quebracho tannin supplementation (Ma et al., 2021). Hydrolytic Chinese gallnut TA also reduced CD of the ileum compared to ZnO diet, as a control (Sun et al., 2021). A lower CD was observed in all intestinal sections of piglets challenged with ETEC in the tannin-supplemented diet group (Zhang et al., 2023).

As a result of changes in VH and CD, weaning impacts the villus height to crypt depth (VH/CD) ratio which is a key parameter for assessing nutrition quality. A high VH/CD ratio indicates healthy gut morphology and good absorption capacity, whereas a low ratio suggests impaired absorption function (Montagne et al., 2003). Supplementation with chestnut tannins positively affected the VH/CD ratio in the duodenum and increased mucosal thickness, reflecting the combined measurement of VH and CD (Bilić-Šobot et al., 2016). Gallnut tannins also increased VH/CD ratio in the duodenum (Yu et al., 2020; Deng et al., 2024; Wang et al., 2020). In the jejunum, this ratio increased following supplementation with TA (Deng et al., 2024) and grape seed proanthocyanidins (Han et al., 2016). Similarly, the VH/CD ratio in the ileum improved with HT from chestnut wood (Liu H. et al., 2020), grape seed extract (Han et al., 2016) and dried grape extract (Rajković et al., 2022). Natural tannin increased VH/CD ratios in duodenum, jejunum and ileum of piglets challenged with ETEC (Zhang et al., 2023). Moreover, diets supplemented with commercial tannin products resulted in a narrower crypt/villi ratio (CD/VH) in the small intestine, meaning that the villi were relatively larger compared to the crypt depth in the intestinal mucosa (Brus et al., 2013a).

The observed differences in the effects of tannins on intestinal morphology reflect the previously discussed structural and functional diversity among tannin types. HT, such as those from chestnut or gallnut, are rapidly hydrolyzed in the upper gastrointestinal tract which may explain their pronounced effects on duodenal VH, CD, and VH/CD ratio (Bilić-Šobot et al., 2016; Song et al., 2021; Deng et al., 2024). In contrast, CT, derived from sources like grape seed or quebracho, are much more resistant to degradation and thus no significant effects on the duodenum have been observed. However, they were shown to influence their morphological effects further along the intestine, particularly in the jejunum and ileum (Han et al., 2016; Ma et al., 2021; Rajković et al., 2022). TA, although classified as a HT, has a relatively simple and uniform molecular structure compared to other HT. This structural simplicity contributes to its strong protein-binding capacity and rapid hydrolysis in the upper gastrointestinal tract. As a result, TA exerts its effects already in duodenum (Wang et al., 2020; Yu et al., 2020), but its effects are highly dose-dependent—low to moderate levels may support epithelial integrity, whereas excessive doses can disrupt villus morphology, particularly in the ileum (Yu et al., 2020).

In the large intestine, which comprises the caecum, colon, and rectum, the primary roles include water and electrolyte absorption, fermentation of undigested material, and feces formation and storage (Szabó et al., 2023). The balance between mitotic and apoptotic cell counts in the large intestine influences epithelial turnover and mucosal health. Chestnut tannin supplementation in fattening boars led to significant, dose-dependent reductions in both apoptotic and mitotic cell counts, particularly in the caecum and descending colon. A notable decrease in apoptotic cell counts was observed in all treatment groups, especially in the ascending colon, where reductions ranged from 16% to 32% depending on the dosage. This decline in apoptotic cells, particularly in the ascending colon, suggests potential improvements in epithelial health following a commercial tannin product supplementation (Bilić-Šobot et al., 2016). In addition, supplementation with quebracho tannins has been shown to reduce colonic mucosa thickness, allowing better water absorption from the stool and lowering the incidence of diarrhea in piglets (Ma et al., 2021).

In summary, supplementation with tannins, particularly from chestnut, gallnut, and grape seed extracts, positively affected gut morphology in pigs by enhancing VH and villus surface area, reducing CD and improving the VH/CD ratio in the small intestine. These effects contribute to better nutrient absorption and overall gut health. Additionally, tannin supplementation in the large intestine led to dose-dependent reductions in mitotic and apoptotic cell counts, indicating improved epithelial integrity, especially in the caecum and colon.

### 4.5 Impact of tannins on intestinal barrier integrity

Maintaining the integrity of the intestinal barrier is crucial for preventing the entry of harmful substances and pathogens to the bloodstream. Tight junction proteins, such as previously mentioned claudins, occludin, and zonula occludens proteins help “seal” the spaces between epithelial cells in the gut, ensuring a selective barrier and controlling the permeability of the intestinal barrier (Hu et al., 2013). Weaning stress associated with inflammation can disrupt tight junction function, leading to a condition known as “leaky gut,” where the intestinal barrier becomes weakened and more susceptible to infections (Wei et al., 2021). Nutritional strategies, such as supplementation with tannins, may mitigate the negative effects of weaning stress and maintain a healthy gut barrier. As shown in Table 5, several studies have demonstrated the impact of different tannin sources on tight junction protein expression in the porcine intestinal epithelium.

**TABLE 5:** Impact of tannins on intestinal barrier integrity.

Source^a^	Name	Protein level	mRNA level	Intestinal segment	Effect^b^	References
Chinese gallnut containing 30% TA	ZO-1	+		Jejunum	↑ expression at 1,000 mg/kg TA (0.1%)	Wang et al. (2022)
Tannalbin, containing 51% TA (from Gallnut)	OCLN	+		Duodenum, Jejunum, Ileum	↑ expression at 1,973.09 mg/kg (0.2%) and 12,004.84 mg/kg (1.0%) TA	Yu et al. (2020)
	OCLN		+	Duodenum	↑expression at 0.2% and 1.0% TA	
	OCLN		+	Jejunum	trend toward ↑ at 1.0% TA	
	ZO-1		+	Ileum	trend toward ↑ at 0.2%	
	ZO-2		+	Duodenum	↑expression at 0.2% TA	
Tannalbin, containing 51% TA (from Gallnut)	ZO-1		+	Jejunum	↑ expression at 0.4% TA	Song et al. (2021)
	ZO-2		+	Jejunum	↑ expression at 0.05%, 0.2%, 0.4% TA	
	CLDN-2		+	Jejunum	↑ expression at 0.2% TA	
Coated tannin (25% tannin content, from chestnut and mainly coated with hydrogenated palm oil)	ZO-1	+		Colon	↑ expression at 1,500 mg/kg (0.15%) CT	Xu et al. (2022)
	CLDN-1	+				
	OCLN	+				
Grape seed proanthocyanidins	OCLN	+		Ileum, Colon	↑ expression at 250 mg/kg GSPs (0.025%)	Han et al. (2016)
Kenwood containing CT	ZO-1		+	Jejunum	↑ expression (in an ETEC strain K88-challenged environment) at 1,000 mg/kg CT (0.1%)	Yi et al. (2023)
	OCLN		+	Ileum		

^a^ TA, tannic acid; CT, condensed tannins.

^b^ ↑, increase.

Differences in tight junction proteins were observed at both the protein and mRNA levels. At the protein level, the supplementation of TA in pig diets has been shown to increase the ZO-1 expression in the jejunum (Wang et al., 2022) and OCLN expression in the duodenum, jejunum, and ileum (Yu et al., 2020). Additionally, coated tannins from chestnut increased ZO-1, OCLN, CLDN-1 protein level in the colon at a dietary inclusion level of 0.15% (Xu et al., 2022). Finally, grape seed proanthocyanidins significantly elevated OCLN expression in the ileal and colonic mucosa of piglets (Han et al., 2016). At the mRNA level, tannin commercial product, which contains TA, was found to increase the expression of tight junction-related genes in the jejunum: ZO-1 in the 0.4% TA group, ZO-2 in all TA groups and CLDN-2 in 0.2% TA group (Song et al., 2021). Both the 0.2% and 1.0% TA diets promoted higher levels of OCLN mRNA in the duodenum. Notably, a 0.2% concentration of tannin was associated with increased ZO-2 mRNA expression levels in the duodenum and a tendency to upregulate ZO-1 mRNA levels in the ileum, while 1.0% tannin content tended to upregulate OCLN mRNA expression levels in the jejunum (Yu et al., 2020). Furthermore, CT enhanced the mRNA expression of ZO-1 in the jejunum and OCLN in the ileum of weaned piglets in an ETEC strain K88-challenged environment (Yi et al., 2023).

Based on these studies, it can be seen that both HT and CT positively affect the levels of tight junction proteins. By doing so, these phytochemicals reduce inflammation, strengthen the integrity of the intestinal barrier, and enhance its protective functions.

### 4.6 Antioxidant, anti-inflammatory, immune modulation and antiparasitic properties of tannins

Due to their unique structure, tannins exhibit other biological effects that are beneficial for pigs during critical weaning and post-weaning periods. Their antioxidant properties are strongly linked to gut health, where they protect the gastrointestinal tract from oxidative damage. This protection may enhance the integrity of intestinal tissues and support the animal’s immune response by reducing inflammation caused by oxidative stress. These effects were summarized in a systematic review that synthesized data from multiple trials and highlighted how tannins from different sources influence the antioxidant status of piglets (Nuamah et al., 2024). Although these antioxidant effects are largely attributed to the tannins chemical structure, particularly those with higher molecular weights showing stronger antioxidant activity, the precise pathways remain unclear and warrant further investigation (Tong et al., 2022).

Tannins are also known for their anti-inflammatory properties. Few studies have examined the impact of tannins on cytokines, which are signaling molecules responsible for regulating inflammation and immune responses. Cytokines such as IL-1β and TNF-α are key mediators of the inflammatory response, and their dysregulation can lead to chronic inflammation and tissue damage. Importantly, TA from gallnut was found to downregulate the pro-inflammatory cytokines IL-1β and TNF-α in the jejunum of weaning pigs (Deng et al., 2024). This suggests that tannins may help modulate the inflammatory processes in the gut, thereby alleviating the inflammatory challenges commonly faced by piglets during the weaning period.

As previously mentioned, the antioxidant and anti-inflammatory activity of tannins are thought to contribute to their immunomodulatory properties; however, the exact mechanisms are not fully understood. Some studies suggest that by reducing oxidative stress and inflammation, tannins help create a more favorable environment for immune cells. This, in turn, promotes the production of immunoglobulins such as IgM and IgG, which are essential for neutralizing pathogens and maintaining immune homeostasis (Liu H. et al., 2020).

Tannins also show promise in the treatment and prevention of parasites in farming. CT, in particular, have been shown to reduce gastrointestinal parasites and improve livestock health. For instance, the consumption of acorn (*Quercus robur*), which is rich in CT, reduced fecal parasite egg counts in Black Slavonian pigs infected with *A. suum* by 96.56%. Similar effects were observed for other gastrointestinal nematodes (*Oesophagostomum* spp., *Strongyloides*, and *Hyostrongylus* spp.), with reductions of up to 93.55% (Rodríguez-Hernández et al., 2023). Tannins effect on worm burdens is likely to protein precipitation on the parasite’s cuticle or digestive tract (Kresˇimir et al., 2004). However, tannins may also act indirectly by stimulating the host’s immune response to the parasite infection as it was shown for grape pomace (Williams et al., 2017).

In summary, tannins exhibit a range of beneficial effects, including antioxidant, anti-inflammatory, anti-parasitic and immune modulating, which can be beneficial in pig farming.

## 5 Mitigation strategies to overcome tannin toxicity

Despite the many potential benefits of tannins, their use in pig nutrition is not without limitations, which has historically led to their classification as anti-nutritional factors (Mueller-Harvey, 2006). One of the main disadvantages of tannins is their astringent nature, which results from their ability to bind with salivary proteins, producing a dry, bitter taste. This in turn can reduce the palatability of feed, leading to lower voluntary feed intake. In piglets, especially during the sensitive post-weaning period, reduced feed consumption can negatively impact growth performance and overall health (Mueller-Harvey, 2006). For example, feeding weaned piglets with black tea extract at dietary inclusion levels of 0.4% and 0.8% led to decreased feed intake and weight gain over a 27-day trial period (Bruins et al., 2011; Orak et al., 2013). Similarly, supplementation of TA at levels of 125–1,000 mg per kilogram of feed resulted in reduction in daily feed intake and average daily gain compared to a control group (0 mg TA/kg diet) (Lee et al., 2010). These findings illustrate the potential drawbacks of tannin supplementation in pig nutrition, emphasizing the need for careful consideration of tannin dosage and the timing of their inclusion in piglet diets.

Another undesirable effect of tannins is their ability to precipitate proteins, inhibit digestive enzymes, and form complexes with minerals, thereby reducing their bioavailability (Jansman, 1993; Girard and Bee, 2020). This in turn can impair growth performance and overall health (Smulikowska et al., 2001; Mueller-Harvey, 2006). For instance, the addition of 0.5% chestnut wood tannins in the feed significantly reduced dry matter and nitrogen digestibility resulting in limited nutrient absorption and disruption of the digestive process in growing pigs (Antongiovanni et al., 2007). Similarly, supplementation of TA at levels of 125–1,000 mg per kilogram of feed resulted in reduced iron absorption, reflected by depleted serum iron concentrations and hemoglobin (Lee et al., 2010). Moreover, compared to low-tannin sorghum (3.7% CT), high-tannin sorghum (54,2% CT) reduced nutrient digestibility in the foregut and fermentation activity in the hindgut of pigs (Pan et al., 2022a; 2022b). In other studies, pigs fed with elevated levels of CT from sorghum showed reduced apparent ileal digestibility of dry matter, crude protein, and amino acids as well as diminished standardized ileal digestibility of amino acids (Mariscal-Landín et al., 2004; Reis de Souza et al., 2019). These decreases in digestibility metrics were predominantly directly proportional to tannin concentration in a diet (Duodu et al., 2003; Mariscal-Landín et al., 2004; Reis de Souza et al., 2019) which implicates its inhibitory effect on digestive enzymes. However, it is worth pointing out that the effects of tannins could have been confounded with the effects of other co-occurring phenols, such as 3-deoxyanthocyanidins and flavan-4-ols (Awika et al., 2005; Dykes et al., 2013). This also might explain why lactating pigs fed sorghum had a poor nitrogen balance (Louis et al., 1991), however further studies will be needed to elucidate that effect.

As mentioned previously, tannins are increasingly recognized for their potential to beneficially modulate gut microbiota composition, e.g., by promoting the growth of beneficial bacterium *Lactobacillus plantarum* (Puljula et al., 2020). Nevertheless, tannins have been primarily known for their antimicrobial properties and are often associated with inhibitory effects on pathogenic microorganisms (McSweeney et al., 2001; Galassi et al., 2019; Liu H. et al., 2020). Therefore, tannin supplementation may negatively influence the gut microbiota composition. For example, an *in vitro* caecal fermentation demonstrated that chestnut wood tannins, at concentrations ranging from 0.75 to 6 g/L, reduced the viable counts of lactobacilli while increasing the populations of potentially pathogenic enterococci and coliforms (Biagi et al., 2010). Moreover, an *in vivo* study in pigs fed tannin-supplemented diets revealed an increase in caecal coliform counts (Biagi et al., 2010). These findings indicate that tannins may exert negative effects on microbial populations under *in vitro* and *in vivo* conditions, potentially disrupting the balance of bacteria in the gut ecosystem.

It is worth noting that tannins are a structurally diverse group and each structural change may alter their impact and interaction of the feed with the host animal (Molino et al., 2023). Therefore, generalizations about the effects and functions of dietary tannins should not rely solely on studies using a single tannin type but rather on those addressing the wide spectrum of tannin chemistry. Unfortunately, many feeding trials have involved a limited number of animals or diets and may have been too short-term to accurately assess the full effects of tannins in animal nutrition. Despite this, the current body of research clearly indicates that tannin toxicity is dose-dependent, with both the concentration and duration of exposure playing critical roles in determining their impact on animal health (Huang et al., 2018). Consequently, the primary strategy for mitigating potential toxicity involves controlling the level and duration of tannin intake. Nevertheless, various processing techniques and mitigation strategies have been developed to limit the negative effects of tannins in feedstuff. Firstly, a selection of extracting agent seems important as studies showed that extraction with aqueous organic solvents such as ethanol (40%), acetone (30%), and methanol (50%) significantly decreased tannins concentrations (Vitti et al., 2005). Optimizing extraction parameters, such as solvent type, concentration, temperature, and extraction time, can therefore play a key role in controlling the tannin content and mitigating tannin-related anti-nutritional effects in animal diets.

In addition to extraction-modifying strategies, physical and chemical processing methods have also been explored as effective strategies to reduce the anti-nutritional effects of tannins in feed. Among physical approaches, milling, soaking, and boiling have been shown to decrease tannin content primarily through leaching or denaturation (Vadivel and Biesalski, 2012; Gupta et al., 2015; Samtiya et al., 2020). Similar effects have also been observed with roasting (Chukwuma et al., 2016). Autoclaving has also been proven highly effective in eliminating antinutrients, however, its practical application is limited by the high cost associated with equipment and energy consumption (Vitti et al., 2005). Chemical treatments represent an alternative to physical processing methods. One commonly proposed strategy is alkaline treatment using agents such as sodium hydroxide, potassium hydroxide or calcium hydroxide (Chavan et al., 1979; Foglia et al., 2022). These treatments reduce tannin content by promoting the oxidation of phenolic compounds at elevated pH levels (Canbolat et al., 2007). Despite their efficacy in neutralizing tannins, the above processes may negatively affect feed quality by reducing palatability and causing the loss of sensitive nutrients, including water-soluble vitamins and minerals, thereby diminishing the overall nutritional value (Chukwuma et al., 2016).

Another approach is the use of tannin-binding agents such as polyvinylpolypyrrolidone (PVPP) and polyethylene glycol (PEG), both of which are synthetic polymers that effectively absorb and precipitate tannins. Additionally, PEG-based detanninification has been found to increase nutrients availability, and decreased microbial inhibition, which in turn led to better animal performance (Makkar, 2003). However, some studies have reported that addition of PEG decrease absorption of amino acids from the intestine, and that its efficacy depends on the protein content of the feed (Waghorn et al., 1987). Another approach that has been tried is the biological degradation of tannins through fermentation by white-rot fungi (Gamble et al., 1996) or lactic acid bacteria (Shang et al., 2019). This approach seems to be the most ecology-compatible however, do not seem to be economically viable mostly due to a prolonged fermentation time measured in days combined with low yields as well as lack of detailed knowledge about the specific enzymes involved in breaking down condensed tannins. Although still in its infancy, fermentation has the potential to find a place in industrial applications in the future. However, significant advancements in enzymatic studies, microbial engineering, and process optimization must be made.

Another strategy to mitigate the negative effects of tannins is the use of different tannin delivery systems. Microencapsulation is a technique that involves enclosing active compounds within small capsules (Kuriokase et al., 2015). This method can be used to mask the astringent and bitter flavors of tannins, thereby improving feed palatability and acceptance in swine diets (Wang et al., 2020). Studies suggest that coating treatments, such as those using hydrogenated palm oil, may also protect tannins from degradation and enhance their effective utilization in animal diets (Xu et al., 2022). Furthermore, microencapsulation enables a controlled release of encapsulated compounds and facilitates their targeted delivery to specific regions of the digestive system, maximizing their benefits and reducing undesirable interactions (Singh et al., 2010).

In summary, mitigation strategies are essential for the effective use of tannin-rich plants in livestock feed, however, the main challenge is to reduce tannin toxicity without compromising their beneficial properties and nutritional value. The focus should be on reducing tannin levels to safe and effective thresholds rather than eliminating them entirely. Excessive removal could strip the feed of tannins’ positive attributes, such as their microbiota-modulating or immunostymulatory properties. Thus, managing the trade-off between tannin reduction and nutrient retention is crucial to optimize feed quality and ensure livestock health and productivity.

## 6 Perspectives

Despite the growing body of research highlighting the potential benefits of tannins in swine nutrition and phytotherapy, several critical knowledge gaps remain that hinder their effective and standardized implementation in pig production systems. Firstly, most existing research primarily focuses on a small selection of commonly used plant sources, such as chestnut, gallnut or quebracho (Caprarulo et al., 2021), limiting our understanding of the full range of tannin effects. This narrow focus may overlook other tannin-rich plant materials, some of which have been reviewed in literature (Fraga-Corral et al., 2021), and which could offer unique bioactive properties or alternative mechanisms of action. Expanding the range of studied sources would allow for a deeper understanding of tannins’ diverse impacts on gut health, antimicrobial activity, and even immune modulation. Moreover, many studies utilize whole plant materials or crude extracts without isolating and characterizing the specific tannins responsible for the effects observed (Han et al., 2016; Reggi et al., 2020; Rajković et al., 2021). This makes it difficult to attribute biological outcomes solely to tannins, as other bioactive compounds present in the extracts may either enhance or mitigate their impact. Therefore, further research on a wider range of plant sources as well as isolated and chemically defined tannins is necessary to determine which molecular structures are responsible for specific eubiotic or anti-nutritional effects. Understanding the structure-activity relationship will be crucial for optimizing their application in targeted nutritional strategies.

In addition to identifying active tannin compounds, the precise mechanisms underlying their eubiotic effects remain insufficiently understood. Although several hypotheses have been presented here—particularly regarding their influence on gut microbiota composition, intestinal barrier integrity, and immune modulation (Liu H. et al., 2020; Yu et al., 2020; Deng et al., 2024)—these require more extensive validation through both *in vivo* and *in vitro* studies. While *in vivo* experiments are essential to comprehensively assess the biological effects and mechanisms of tannins in living organisms, *in vitro* models are often favored due to their practicality, cost-efficiency, and ethical advantages. However, it is important to acknowledge that research on the effects of tannins in pig nutrition has demonstrated that outcomes can differ between *in vitro* and *in vivo* experiments (Biagi et al., 2010; Puljula et al., 2020). These discrepancies are largely due to the complexity of biological systems in living organisms, where interactions between dietary compounds, host physiology, and microbiota cannot be fully replicated *in vitro*. Consequently, a balanced approach using both *in vitro* and *in vivo* models is necessary to gain a comprehensive understanding of tannins’ effects. Advanced experimental designs and technologies, such as organ-on-a-chip and omics approaches, could help bridge the gap between these models and provide more accurate insights into tannins biological mechanisms.

In parallel, understanding how genetic factors influence the response to dietary tannins is essential for improving pig nutrition and management strategies. Although research in this area is still limited, recent studies have identified genetic factors that affect how different pig breeds detect and metabolize tannin-rich feed components. Scientists merged blood metabolite profiling and genetic mapping in over 1,300 pigs (Large White and Duroc) to identify 97 metabolomics-genomics links associated with 126 metabolites. These findings show that specific gene variants affect nutrient metabolism, including plant compounds like tannins, by affecting enzymes, transporters, and metabolic pathways (Bovo et al., 2025). Breed-specific differences has also been observed in taste and nutrient-sensing genes, which can influence pigs ability and willingness to consume tannin-rich diets. In Iberian pigs, the downregulation of taste receptor genes, particularly TAS2Rs, TAS1Rs, and fatty acid receptors, suggests a reduced sensitivity to taste, likely linked to their traditional, tannin-rich diet. This lower taste sensitivity may help explain their higher voluntary feed intake compared to Duroc pigs, which show higher expression of these genes and more selective feeding behavior (Óvilo et al., 2025). In addition, pigs fed tannin-rich diets show parotid gland hypertrophy and increased secretion of basic and acidic proline-rich proteins - a genetically driven adaptive response. Notably, Iberian pigs, known for their rustic heritage, exhibit a strong ability to neutralize tannins through increased secretion of proline-rich proteins, enabling them to tolerate even high dietary tannin levels (Mavri et al., 2022). These findings highlight the importance of genetic background in shaping both the physiological and behavioral responses of pigs to dietary tannins, offering valuable insights for breed-specific feeding strategies. However, current studies have focused on a limited number of breeds, underscoring the need for broader comparative research across diverse pig populations.

Other significant issue is a lack of long-term and multi-generational studies evaluating the impact of tannins on overall pig health. Without long-term studies, it remains unclear whether the observed positive effects of tannins in the short term are sustainable or if they may have unintended negative consequences over extended periods. Chronic exposure to tannins could potentially influence host physiology and gut microbiota stability over time, potentially leading to adaptive microbial shifts or resistance mechanisms. Multi-generational exposure could potentially have effects on reproductive performance, offspring development, immune system programming, and epigenetic modifications altering susceptibility to diseases or metabolic traits in subsequent generations. Notably, the breed-specific differences in tannin tolerance described above—such as variation in taste receptor expression and salivary protein secretion—suggest that certain adaptive responses could potentially develop with prolonged exposure to tannin-rich diets. Studies in ruminants further support this idea, indicating that adaptations such as shifts in the rumen microbiota and increased production of tannin-binding salivary proteins can develop over time, enabling greater dietary tolerance (Smith et al., 2005; Vargas-Magaña et al., 2013). Pigs, being monogastric, lack a rumen, and thus the same microbial mechanisms may not apply; however, the possibility of host-driven adaptation across generations cannot be ruled out. Therefore, a more comprehensive, long-term, and multi-generational research approach is essential to fully understand the implications of tannin use in pig nutrition and to ensure their efficacy and safety in the long term.

Another important limitation is the small amount of available information regarding how tannins interact with different pig diets, as well as the lack of research on appropriate tannin inclusion levels across various growth stages. The interaction of tannins with other dietary components, such as fiber, protein, and minerals, remains poorly understood, yet is crucial for optimizing diet formulations aimed at promoting gut health and overall physiological performance. Future research should focus on evaluating these interactions to determine the most effective dietary compositions. In addition, studies should consider adjusting tannin inclusion levels according to the pigs’ growth stage, as nutritional needs and gut sensitivity vary throughout development. Tailoring tannin levels to specific physiological phases may help maximize benefits while minimizing potential negative effects.

Beyond biological efficacy, practical considerations must also be addressed. These include cost-effectiveness, processing stability, and acceptance by farmers and feed manufacturers. Even the most biologically promising compounds may face barriers to adoption if they are not economically viable or compatible with existing feed processing technologies. Therefore, the economic feasibility of tannin-based feed additives, including production costs, return on investment, and potential savings from reduced antibiotic use, should be thoroughly evaluated. Additionally, strategies for effective integration into commercial pig diets must be developed, taking into account formulation compatibility and user acceptance. Bridging the gap between scientific research and industry application will be essential for the widespread adoption of tannins as functional feed ingredients.

Finally, regulatory aspects must be addressed. Despite increasing interest from veterinarians and livestock producers in herbal medicinal products and feed supplements, their availability on the veterinary market still remains limited. This situation is largely due to a lack of harmonized regulatory frameworks and clear guidelines for the market authorization of herbal medicinal products for veterinary use in the EU and internationally. The implementation of EU Regulation 2019/6, while aiming to modernize veterinary medicinal product regulation, has not sufficiently addressed herbal medicinal products requirements, leaving regulatory uncertainty that hinders product development and commercialization. These gaps are accompanied by the need for comprehensive safety and efficacy assessments not only for animal health, but also for food safety in products derived from treated livestock. To fully realize the potential of tannin-containing plant materials in promoting gut health and reducing synthetic antimicrobial or antibiotic use in livestock, it is essential to establish a clear legal definition for herbal medicinal products for veterinary use, along with harmonized quality control measures and safety standards. Moreover, the development of authoritative, species-specific monographs on veterinary medicinal plant products with validated therapeutic indications would support evidence-based product development and the competitiveness of industry in Europe and globally.

To summarize, despite promising evidence supporting the use of tannins in pig nutrition, key knowledge gaps remain. Current research focuses on only a few plant sources and lacks clarity regarding the active compounds and their mechanisms of action. Genetic differences between pig breeds suggest a potential for adaptive responses to tannins, but long-term and multi-generational studies are needed to confirm these effects. Additionally, the interaction of tannins with other dietary components and the determination of appropriate inclusion levels across growth stages require further investigation. Practical and regulatory barriers—such as economic feasibility, processing stability, and unclear legislation—must also be addressed to enable the safe and effective adoption of tannin-based strategies in swine production. Notably, due to the close anatomical and physiological similarities between the porcine and human gastrointestinal tracts, pigs are widely recognized as a relevant translational model. Therefore, advances in understanding tannins’ effects in pigs may also offer insights relevant to human health and support the development of tannin-based eubiotic strategies in medicine.

## 7 Conclusion

Traditional strategies to reduce post-weaning intestinal dysfunction in piglets have focused on dietary interventions, such as the use of antibiotics or dietary minerals like zinc oxide. However, recent studies highlight the potential of natural compounds, such as tannins, as effective alternatives, aiming to eliminate the use of antibiotics in pig production. From an eubiotic perspective, the strategic use of tannins in pig nutrition and phytotherapy offers a promising approach for enhancing microbial balance and improving gut health. Plant-derived tannins exhibit antimicrobial, anti-inflammatory, antioxidant, and immuno-modulatory properties that align with the goals of eubiotic feeding. While further research is needed to optimize their application, current evidence supports their potential as valuable tools in pig production systems. The combined benefits of improved animal welfare, environmental sustainability, and economic return make tannins an attractive and responsible choice for modern animal production systems.

## References

[B1] Aguilar-Galvez A. Noratto G. Chambi F. Debaste F. Campos D. (2014). Potential of tara (*Caesalpinia spinosa*) gallotannins and hydrolysates as natural antibacterial compounds. Food Chem. 156, 301–304. 10.1016/j.foodchem.2014.01.110 24629972

[B2] Alejo‐Armijo A. Glibota N. Frías M. P. Altarejos J. Gálvez A. Ortega‐Morente E. (2017). Antimicrobial and antibiofilm activities of procyanidins extracted from laurel wood against a selection of foodborne microorganisms. Int. J. Food Sci. Tech. 52, 679–686. 10.1111/ijfs.13321

[B3] Alshaibani D. Zhang R. Wu V. C. H. (2017). Antibacterial characteristics and activity of *Vaccinium macrocarpon* proanthocyanidins against diarrheagenic *Escherichia coli* . J. Funct. Foods 39, 133–138. 10.1016/j.jff.2017.10.003

[B4] Antongiovanni M. Minieri S. Petacchi F. (2007). Effect of tannin supplementation on nitrogen digestibility and retention in growing pigs. Ital. J. Anim. Sci. 6, 245–247. 10.4081/ijas.2007.1s.245

[B5] Awika J. M. Rooney L. W. Waniska R. D. (2005). Anthocyanins from black sorghum and their antioxidant properties. Food Chem. 90, 293–301. 10.1016/j.foodchem.2004.03.058

[B6] Bag A. Bhattacharyya S. K. Chattopadhyay R. R. (2013). Isolation and identification of a gallotannin 1,2,6-tri- *O* -galloyl- **β** - d -glucopyranose from hydroalcoholic extract of *Terminalia chebula* fruits effective against multidrug-resistant uropathogens. J. Appl. Microbiol. 115, 390–397. 10.1111/jam.12256 23683054

[B7] Bahelka I. Stupka R. Čítek J. Šprysl M. Bučko O. Fľak P. (2023). Eating quality of pork from entire male pigs after dietary supplementation with hydrolysable tannins. Animals 13, 893. 10.3390/ani13050893 36899752 PMC10000101

[B8] Bednorz C. Oelgeschläger K. Kinnemann B. Hartmann S. Neumann K. Pieper R. (2013). The broader context of antibiotic resistance: zinc feed supplementation of piglets increases the proportion of multi-resistant *Escherichia coli in vivo* . Int. J. Med. Microbiol. 303, 396–403. 10.1016/j.ijmm.2013.06.004 23856339

[B9] Bee G. Silacci P. Ampuero-Kragten S. Čandek-Potokar M. Wealleans A. L. Litten-Brown J. (2017). Hydrolysable tannin-based diet rich in gallotannins has a minimal impact on pig performance but significantly reduces salivary and bulbourethral gland size. Animal 11, 1617–1625. 10.1017/S1751731116002597 28004617 PMC5561437

[B10] Biagi G. Cipollini I. Paulicks B. R. Roth F. X. (2010). Effect of tannins on growth performance and intestinal ecosystem in weaned piglets. Arch. Anim. Nutr. 64, 121–135. 10.1080/17450390903461584 20481351

[B11] Bilić-Šobot D. Kubale V. Škrlep M. Čandek-Potokar M. Prevolnik Povše M. Fazarinc G. (2016). Effect of hydrolysable tannins on intestinal morphology, proliferation and apoptosis in entire male pigs. Arch. Anim. Nutr. 70, 378–388. 10.1080/1745039X.2016.1206735 27434497

[B12] Boakye Y. D. (2016). Anti-infective properties and time-kill kinetics of *Phyllanthus muellerianus* and its major constituent, geraniin. Med. Chem. 6. 10.4172/2161-0444.1000332

[B13] Bottegal D. N. Latorre M. Á. Lobón S. Verdú M. Álvarez-Rodríguez J. (2024). Fattening pigs with tannin-rich source (*Ceratonia siliqua* L.) and high doses of vitamin E: effects on growth performance, economics, digestibility, physiology, and behaviour. Animals 14, 1855. 10.3390/ani14131855 38997967 PMC11240671

[B14] Bovo S. Ribani A. Fanelli F. Galimberti G. Martelli P. L. Trevisi P. (2025). Merging metabolomics and genomics provides a catalog of genetic factors that influence molecular phenotypes in pigs linking relevant metabolic pathways. Genet. Sel. Evol. 57, 11. 10.1186/s12711-025-00960-8 40050712 PMC11887101

[B15] Brosnahan A. J. Brown D. R. (2012). Porcine IPEC-J2 intestinal epithelial cells in microbiological investigations. Vet. Microbiol. 156, 229–237. 10.1016/j.vetmic.2011.10.017 22074860 PMC3289732

[B16] Bruins M. J. Vente-Spreeuwenberg M. a. M. Smits C. H. Frenken L. G. J. (2011). Black tea reduces diarrhoea prevalence but decreases growth performance in enterotoxigenic *Escherichia coli*-infected post-weaning piglets. J. Anim. Physiol. Anim. Nutr. Berl. 95, 388–398. 10.1111/j.1439-0396.2010.01066.x 21039929

[B17] Brus M. Bavdek S. Skok J. Škorjanc D. (2013a). Impact of supplementing pig diet with tannins on histological characteristics of small intestine and growth performance of fattening pigs. Acta Agriculturae Slovenica. Suplement 4, 135–138. 10.14720/aas-s.2013.4.19037

[B18] Brus M. DolinŠek J. DolinŠeK J. CenCiČ A. (2013b). Effect of chestnut (*Castanea sativa* Mill.) wood tannins and organic acids on growth performance and faecal microbiota of pigs from 23 to 127 days of age. Bulg. J. Agric. Sci. 19, 841–847.

[B19] Brus M. Langerholc T. Škorjanc D. (2013c). Effect of hydrolysable tannins on proliferation of small intestinal porcine and human enterocytes. Acta Agriculturae Slovenica. Suplement 4, 131–134. 10.14720/aas-s.2013.4.19036

[B20] Canbolat O. Ozkan C. O. Kamalak A. (2007). Effects of NaOH treatment on condensed tannin contents and gas production kinetics of tree leaves. Anim. Feed Sci. Technol. 138, 189–194. 10.1016/j.anifeedsci.2007.06.024

[B21] Canibe N. Højberg O. Kongsted H. Vodolazska D. Lauridsen C. Nielsen T. S. (2022). Review on preventive measures to reduce post-weaning diarrhoea in piglets. Animals (Basel) 12, 2585. 10.3390/ani12192585 36230326 PMC9558551

[B22] Cappai M. G. Wolf P. Pinna W. Kamphues J. (2013). Pigs use endogenous proline to cope with acorn (*Quercus pubescens Willd*.) combined diets high in hydrolysable tannins. Livest. Sci. 155, 316–322. 10.1016/j.livsci.2013.05.003

[B23] Caprarulo V. Hejna M. Giromini C. Liu Y. Dell’Anno M. Sotira S. (2020). Evaluation of dietary administration of chestnut and quebracho tannins on growth, serum metabolites and fecal parameters of weaned piglets. Animals (Basel) 10, 1945. 10.3390/ani10111945 33105748 PMC7690424

[B24] Caprarulo V. Giromini C. Rossi L. (2021). Review: chestnut and quebracho tannins in pig nutrition: the effects on performance and intestinal health. Animal 15, 100064. 10.1016/j.animal.2020.100064 33516022

[B25] Chavan J. K. Kadam S. S. Ghonsikar C. P. Salunkhe D. K. (1979). Removal of tannins and improvement of *in vitro* protein digestibility of sorghum seeds by soaking in alkali. J. Food Sci. 44, 1319–1322. 10.1111/j.1365-2621.1979.tb06429.x

[B26] Chukwuma O. E. Taiwo O. O. Boniface U. V. (2016). Effect of the traditional cooking methods (boiling and roasting) on the nutritional profile of quality protein maize. J. Food Nutr. Sci. 4, 34–40. 10.11648/j.jfns.20160402.12

[B27] Clemente J. C. Ursell L. K. Parfrey L. W. Knight R. (2012). The impact of the gut microbiota on human health: an integrative view. Cell 148, 1258–1270. 10.1016/j.cell.2012.01.035 22424233 PMC5050011

[B28] Coddens A. Loos M. Vanrompay D. Remon J. P. Cox E. (2017). Cranberry extract inhibits *in vitro* adhesion of F4 and F18 + *Escherichia coli* to pig intestinal epithelium and reduces *in vivo* excretion of pigs orally challenged with F18 + verotoxigenic *E. coli* . Vet. Microbiol. 202, 64–71. 10.1016/j.vetmic.2017.01.019 28161211

[B29] Costa M. M. Alfaia C. M. Lopes P. A. Pestana J. M. Prates J. A. M. (2022). Grape by-products as feedstuff for pig and poultry production. Animals (Basel) 12, 2239. 10.3390/ani12172239 36077957 PMC9454619

[B30] Costabile A. Sanghi S. Martín-Pelaez S. Mueller-Harvey I. Gibson G. R. Rastall R. A. (2011). Inhibition of *Salmonella* typhimurium by tannins *in vitro* . J. Food Agric. Environ. 9, 119–124.

[B31] Deng Z. Wang J. Wang J. Yan Y. Huang Y. Chen C. (2024). Tannic acid extracted from gallnut improves intestinal health with regulation of redox homeostasis and gut microbiota of weaned piglets. Anim. Res. One Health. 2, 16–27. 10.1002/aro2.51

[B32] Ding Z. Mo M. Zhang K. Bi Y. Kong F. (2021). Preparation, characterization and biological activity of proanthocyanidin-chitosan nanoparticles. Int. J. Biol. Macromol. 188, 43–51. 10.1016/j.ijbiomac.2021.08.010 34364936

[B33] Duan H. Yu L. Tian F. Zhai Q. Fan L. Chen W. (2022). Antibiotic-induced gut dysbiosis and barrier disruption and the potential protective strategies. Crit. Rev. Food Sci. Nutr. 62, 1427–1452. 10.1080/10408398.2020.1843396 33198506

[B34] Duodu K. G. Taylor J. R. N. Belton P. S. Hamaker B. R. (2003). Factors affecting sorghum protein digestibility. J. Cereal Sci. 38, 117–131. 10.1016/S0733-5210(03)00016-X

[B35] Dykes L. Rooney W. L. Rooney L. W. (2013). Evaluation of phenolics and antioxidant activity of black sorghum hybrids. J. Cereal Sci. 58, 278–283. 10.1016/j.jcs.2013.06.006

[B36] Engels C. Schieber A. Gänzle M. G. (2011). Inhibitory spectra and modes of antimicrobial action of gallotannins from mango kernels (*Mangifera indica* L.). Appl. Environ. Microbiol. 77, 2215–2223. 10.1128/AEM.02521-10 21317249 PMC3067452

[B37] Espín J. C. González-Barrio R. Cerdá B. López-Bote C. Rey A. I. Tomás-Barberán F. A. (2007). Iberian pig as a model to clarify obscure points in the bioavailability and metabolism of ellagitannins in humans. J. Agric. Food Chem. 55, 10476–10485. 10.1021/jf0723864 17990850

[B38] Foglia F. Drapcho C. Nghiem J. (2022). Effectiveness of tannin removal by alkaline pretreatment on sorghum ethanol production. Fermentation 8, 274. 10.3390/fermentation8060274

[B39] Fraga-Corral M. Otero P. Cassani L. Echave J. Garcia-Oliveira P. Carpena M. (2021). Traditional applications of tannin rich extracts supported by scientific data: chemical composition, bioavailability and bioaccessibility. Foods 10, 251. 10.3390/foods10020251 33530516 PMC7912241

[B40] Galassi G. Mason F. Rapetti L. Crovetto G. M. Spanghero M. (2019). Digestibility and metabolic utilisation of diets containing chestnut tannins and their effects on growth and slaughter traits of heavy pigs. J. Anim. Sci. 18, 746–753. 10.1080/1828051X.2019.1570361 24243899

[B41] Gamble G. R. Akin D. E. Makkar H. P. Becker K. (1996). Biological degradation of tannins in sericea lespedeza (*Lespedeza cuneata*) by the white rot fungi *Ceriporiopsis subvermispora* and *Cyathus stercoreus* analyzed by solid-state 13C nuclear magnetic resonance spectroscopy. Appl. Environ. Microbiol. 62, 3600–3604. 10.1128/aem.62.10.3600-3604.1996 8837414 PMC168166

[B42] Geng S. Yang L. Cheng F. Zhang Z. Li J. Liu W. (2020). Gut microbiota are associated with psychological stress-induced defections in intestinal and blood–brain barriers. Front. Microbiol. 10, 3067. 10.3389/fmicb.2019.03067 32010111 PMC6974438

[B43] Girard M. Bee G. (2020). Invited review: tannins as a potential alternative to antibiotics to prevent coliform diarrhea in weaned pigs. Animal 14, 95–107. 10.1017/S1751731119002143 31571564

[B44] Giurazza R. Mazza M. C. Andini R. Sansone P. Pace M. C. Durante-Mangoni E. (2021). Emerging treatment options for multi-drug-resistant bacterial infections. Life (Basel) 11, 519. 10.3390/life11060519 34204961 PMC8229628

[B45] Gontijo D. C. Gontijo P. C. Brandão G. C. Diaz M. A. N. De Oliveira A. B. Fietto L. G. (2019). Antioxidant study indicative of antibacterial and antimutagenic activities of an ellagitannin-rich aqueous extract from the leaves of *Miconia latecrenata* . J. Ethnopharmacol. 236, 114–123. 10.1016/j.jep.2019.03.007 30853643

[B46] Granica S. Vahjen W. Zentek J. Melzig M. F. Pawłowska K. A. Piwowarski J. P. (2020). *Lythrum salicaria* ellagitannins stimulate IPEC-J2 cells monolayer formation and inhibit enteropathogenic *Escherichia coli* growth and adhesion. J. Nat. Prod. 83, 3614–3622. 10.1021/acs.jnatprod.0c00776 33270444 PMC7771025

[B47] Gupta R. K. Gangoliya S. S. Singh N. K. (2015). Reduction of phytic acid and enhancement of bioavailable micronutrients in food grains. J. Food Sci. Technol. 52, 676–684. 10.1007/s13197-013-0978-y 25694676 PMC4325021

[B48] Gurning K. Simanjuntak H. A. Purba H. Situmorang R. F. R. Barus L. Silaban S. (2021). Determination of total tannins and antibacterial activities ethanol extraction seri (*Muntingia calabura* L.) leaves. J. Phys. Conf. Ser. 1811, 012121. 10.1088/1742-6596/1811/1/012121

[B49] Han M. Song P. Huang C. Rezaei A. Farrar S. Brown M. A. (2016). Dietary grape seed proanthocyanidins (GSPs) improve weaned intestinal microbiota and mucosal barrier using a piglet model. Oncotarget 7, 80313–80326. 10.18632/oncotarget.13450 27880936 PMC5348322

[B50] Hancock V. Dahl M. Vejborg R. M. Klemm P. (2010). Dietary plant components ellagic acid and tannic acid inhibit *Escherichia coli* biofilm formation. J. Med. Microbiol. 59, 496–498. 10.1099/jmm.0.013680-0 19959627

[B51] Haslam E. (1996). Natural polyphenols (vegetable tannins) as drugs: possible modes of action. J. Nat. Prod. 59, 205–215. 10.1021/np960040+ 8991956

[B52] Heo J. M. Opapeju F. O. Pluske J. R. Kim J. C. Hampson D. J. Nyachoti C. M. (2013). Gastrointestinal health and function in weaned pigs: a review of feeding strategies to control post‐weaning diarrhoea without using in‐feed antimicrobial compounds. J. Anim. Physiol. Anim. Nutr. Berl. 97, 207–237. 10.1111/j.1439-0396.2012.01284.x 22416941

[B53] Hossain M. M. Cho S. B. Kim I. H. (2024). Strategies for reducing noxious gas emissions in pig production: a comprehensive review on the role of feed additives. J. Anim. Sci. Technol. 66, 237–250. 10.5187/jast.2024.e15 38628679 PMC11016746

[B54] Hu C. H. Xiao K. Luan Z. S. Song J. (2013). Early weaning increases intestinal permeability, alters expression of cytokine and tight junction proteins, and activates mitogen-activated protein kinases in pigs. J. Anim. Sci. 91, 1094–1101. 10.2527/jas.2012-5796 23230104

[B55] Huang Q. Liu X. Zhao G. Hu T. Wang Y. (2018). Potential and challenges of tannins as an alternative to in-feed antibiotics for farm animal production. Anim. Nutr. 4, 137–150. 10.1016/j.aninu.2017.09.004 30140753 PMC6104569

[B56] Itza-Ortiz M. Segura-Correa J. Parra-Suescún J. Aguilar-Urquizo E. Escobar-Gordillo N. Itza-Ortiz M. (2019). Correlation between body weight and intestinal villi morphology in finishing pigs. Acta Univ. 29, 1–7. 10.15174/au.2019.2354

[B57] Jansman A. J. M. (1993). Tannins in feedstuffs for simple-stomached animals. Nutr. Res. Rev. 6, 209–236. 10.1079/NRR19930013 19094309

[B58] Khanbabaee K. van Ree T. (2001). Tannins: classification and definition. Nat. Prod. Rep. 18, 641–649. 10.1039/b101061l 11820762

[B59] Kim K. Song M. Liu Y. Ji P. (2022). Enterotoxigenic *Escherichia coli* infection of weaned pigs: intestinal challenges and nutritional intervention to enhance disease resistance. Front. Immunol. 13, 885253. 10.3389/fimmu.2022.885253 35990617 PMC9389069

[B60] Kiss A. K. Piwowarski J. P. (2018). Ellagitannins, gallotannins and their metabolites - the contribution to the anti-inflammatory effect of food products and medicinal plants. Curr. Med. Chem. 25, 4946–4967. 10.2174/0929867323666160919111559 27655073

[B61] Klug T. V. Novello J. Laranja D. C. Aguirre T. A. S. De Oliveira Rios A. Tondo E. C. (2017). Effect of tannin extracts on biofilms and attachment of *Escherichia coli* on lettuce leaves. Food Bioprocess Technol. 10, 275–283. 10.1007/s11947-016-1812-0

[B62] Koo H. Allan R. N. Howlin R. P. Stoodley P. Hall-Stoodley L. (2017). Targeting microbial biofilms: current and prospective therapeutic strategies. Nat. Rev. Microbiol. 15, 740–755. 10.1038/nrmicro.2017.99 28944770 PMC5685531

[B63] Kresˇimir S. Karolyi D. Beck R. Goran K. Vicković I. Đikić M. (2004). Effect of acorn (*Quercus robur*) intake on faecal egg count in outdoor reared black Slavonian pig. Acta Agricul Slov. Suppl. 1, 173–178. 10.14720/aas-s.2004.1.19422

[B64] Kuriokase B. Padma S. Priya S. P. (2015). A review on microcapsules. Glob. J. Pharmacol. 10.5829/idosi.gjp.2015.9.1.91110

[B65] Lee S. H. Shinde P. L. Choi J. Y. Kwon I. K. Lee J. K. Pak S. I. (2010). Effects of tannic acid supplementation on growth performance, blood hematology, iron status and faecal microflora in weanling pigs. Livest. Sci. 131, 281–286. 10.1016/j.livsci.2010.04.013

[B66] Lee H. J. Choi I. H. Kim D. H. Amanullah S. M. Kim S. C. (2016). Nutritional characterization of tannin rich chestnut (*Castanea*) and its meal for pig. J. Appl. Anim. Res. 44, 258–262. 10.1080/09712119.2015.1031779

[B67] Li N. Luo M. Fu Y. Zu Y. Wang W. Zhang L. (2013). Effect of corilagin on membrane permeability of *Escherichia coli, Staphylococcus aureus* and *Candida albicans* . Phytotherapy Res. 27, 1517–1523. 10.1002/ptr.4891 23192753

[B68] Li G. Yan C. Xu Y. Feng Y. Wu Q. Lv X. (2014). Punicalagin inhibits *Salmonella* virulence factors and has anti-*quorum*-sensing potential. Appl. Environ. Microbiol. 80, 6204–6211. 10.1128/AEM.01458-14 25085489 PMC4178673

[B69] Li Q. H. Yan H. S. Li H. Q. Gao J. J. Hao R. R. (2020). Effects of dietary supplementation with grape seed procyanidins on nutrient utilisation and gut function in weaned piglets. Animal 14, 491–498. 10.1017/S1751731119002234 31588892

[B70] Li M. Wang A. Zhang Y. Han T. Guan L. Fan D. (2022). A comprehensive review on ethnobotanical, phytochemical and pharmacological aspects of *Rhus chinensis* Mill. J. Ethnopharmacol. 293, 115288. 10.1016/j.jep.2022.115288 35430289

[B71] Liu W. Guo K. (2024). Tannic acid alleviates ETEC K88 ‐induced intestinal damage through regulating the p62‐keap1‐Nrf2 and TLR4‐NF‐κB‐NLRP3 pathway in IPEC‐J2 cells. J. Sci. Food Agric. 104, 5186–5196. 10.1002/jsfa.13343 38288747

[B72] Liu H. Hu J. Mahfuz S. Piao X. (2020a). Effects of hydrolysable tannins as zinc oxide substitutes on antioxidant status, immune function, intestinal morphology, and digestive enzyme activities in weaned piglets. Animals (Basel) 10, 757. 10.3390/ani10050757 32349238 PMC7277717

[B73] Liu L. Ge C. Zhang Y. Ma W. Su X. Chen L. (2020b). Tannic acid-modified silver nanoparticles for enhancing anti-biofilm activities and modulating biofilm formation. Biomater. Sci. 8, 4852–4860. 10.1039/D0BM00648C 32734981

[B74] Louis G. F. Lewis A. J. Peo E. R. (1991). Feeding value of grain sorghum for the lactating sow. J. Anim. Sci. 69, 223–229. 10.2527/1991.691223x 2005018

[B75] Luecke R. W. Thorp F. Newland H. W. Mcmillen W. N. (1951). The growth promoting effects of various antibiotics on pigs. J. Anim. Sci. 10, 538–542. 10.2527/jas1951.102538x 14832161

[B76] Ma Y. Wang Y. Zhang H. Sun W. Li Z. Zhang F. (2020). Antimicrobial mechanism of strictinin isomers extracted from the root of *Rosa roxburghii* Tratt (Ci Li Gen). J. Ethnopharmacol. 250, 112498. 10.1016/j.jep.2019.112498 31877366

[B77] Ma M. Chambers J. K. Uchida K. Ikeda M. Watanabe M. Goda Y. (2021). Effects of supplementation with a quebracho tannin product as an alternative to antibiotics on growth performance, diarrhea, and overall health in early-weaned piglets. Animals (Basel) 11, 3316. 10.3390/ani11113316 34828046 PMC8614404

[B78] Ma M. Enomoto Y. Takahashi T. Uchida K. Chambers J. K. Goda Y. (2024). Study of the effects of condensed tannin additives on the health and growth performance of early-weaned piglets. Animals (Basel) 14, 2337. 10.3390/ani14162337 39199871 PMC11350907

[B79] Mailoa M. N. Mahendradatta M. Laga A. Djide N. (2014). Antimicrobial activities of tannins extract from guava leaves (*Psidium Guajava* L) on pathogens microbial. Int. J. Sci. and Technol. Res. 3, 236–241.

[B80] Makkar H. P. S. (2003). Effects and fate of tannins in ruminant animals, adaptation to tannins, and strategies to overcome detrimental effects of feeding tannin-rich feeds. Small Rumin. Res. 49, 241–256. 10.1016/S0921-4488(03)00142-1

[B81] Mariscal-Landín G. Avellaneda J. H. Reis de Souza T. C. Aguilera A. Borbolla G. A. Mar B. (2004). Effect of tannins in sorghum on amino acid ileal digestibility and on trypsin (E.C.2.4.21.4) and chymotrypsin (E.C.2.4.21.1) activity of growing pigs. Anim. Feed Sci. Technol. 117, 245–264. 10.1016/j.anifeedsci.2004.09.001

[B82] Mavri M. Čandek-Potokar M. Fazarinc G. Škrlep M. Rutland C. S. Potočnik B. (2022). Salivary gland adaptation to dietary inclusion of hydrolysable tannins in boars. Animals (Basel) 12, 2171. 10.3390/ani12172171 36077892 PMC9454789

[B83] McSweeney C. S. Palmer B. McNeill D. M. Krause D. O. (2001). Microbial interactions with tannins: nutritional consequences for ruminants. Anim. Feed Sci. Technol. 91, 83–93. 10.1016/S0377-8401(01)00232-2

[B84] Mehansho H. Butler L. G. Carlson D. M. (1987). Dietary tannins and salivary proline-rich proteins: interactions, induction, and defense mechanisms. Annu. Rev. Nutr. 7, 423–440. 10.1146/annurev.nu.07.070187.002231 3038154

[B85] Mertenat D. Cero M. D. Vogl C. R. Ivemeyer S. Meier B. Maeschli A. (2020). Ethnoveterinary knowledge of farmers in bilingual regions of Switzerland - is there potential to extend veterinary options to reduce antimicrobial use? J. Ethnopharmacol. 246, 112184. 10.1016/j.jep.2019.112184 31465817 PMC7185669

[B86] Miragoli F. Patrone V. Prandini A. Sigolo S. Dell’Anno M. Rossi L. (2021). A mixture of quebracho and chestnut tannins drives butyrate-producing bacteria populations shift in the gut microbiota of weaned piglets. PLoS One 16, e0250874. 10.1371/journal.pone.0250874 33914832 PMC8084250

[B87] Mirelle R. Patrice D. N. Josaphat N. (2016). Evaluation of the antimicrobial activity of tannin extracted from the barks of *Erythrophleum guineensis* (Caesalpiniaceae). J. Pharmacogn. Phytochem. 5, 287–291.

[B88] Mnisi C. M. Njeri F. M. Maina A. N. Waliaula P. K. Cheng V. Kumalo I. (2025). A review on the potential use of eubiotics in non-chicken poultry species. Trop. Anim. Health Prod. 57, 213. 10.1007/s11250-025-04466-9 40335869 PMC12058837

[B89] Molino S. Pilar Francino M. Ángel Rufián Henares J. (2023). Why is it important to understand the nature and chemistry of tannins to exploit their potential as nutraceuticals? Food Res. Int. 173, 113329. 10.1016/j.foodres.2023.113329 37803691

[B90] Molotla-Torres D. E. Guzmán-Mejía F. Godínez-Victoria M. Drago-Serrano M. E. (2023). Role of stress on driving the intestinal paracellular permeability. Curr. Issues Mol. Biol. 45, 9284–9305. 10.3390/cimb45110581 37998758 PMC10670774

[B91] Montagne L. Pluske J. R. Hampson D. J. (2003). A review of interactions between dietary fibre and the intestinal mucosa, and their consequences on digestive health in young non-ruminant animals. Anim. Feed Sci. Technol. 108, 95–117. 10.1016/S0377-8401(03)00163-9

[B92] Mueller-Harvey I. (2006). Unravelling the conundrum of tannins in animal nutrition and health. J. Sci. Food Agric. 86, 2010–2037. 10.1002/jsfa.2577

[B93] Munengwa A. Nyahangare E. T. Jambwa P. Mugoti A. Mandara S. McGaw L. J. (2025). Ethnoveterinary medicines used by smallholder farmers for treatment of goat ailments in Chikomba, Murewa, Gutu and Mwenezi districts of Zimbabwe: is there cultural consensus in use practices? J. Ethnopharmacol. 342, 119324. 10.1016/j.jep.2025.119324 39855433

[B94] Myrie S. B. Bertolo R. F. Sauer W. C. Ball R. O. (2008). Effect of common antinutritive factors and fibrous feedstuffs in pig diets on amino acid digestibilities with special emphasis on threonine. J. Anim. Sci. 86, 609–619. 10.2527/jas.2006-793 17998420

[B95] Nie H.-Y. Ge J. Huang G.-X. Liu K.-G. Yue Y. Li H. (2024). New insights into the intestinal barrier through “gut-organ” axes and a glimpse of the microgravity’s effects on intestinal barrier. Front. Physiol. 15, 1465649. 10.3389/fphys.2024.1465649 39450142 PMC11499591

[B96] Nowak P. Kasprowicz-Potocka M. Zaworska A. Nowak W. Stefańska B. Sip A. (2017). The effect of eubiotic feed additives on the performance of growing pigs and the activity of intestinal microflora. Arch. Anim. Nutr. 71, 455–469. 10.1080/1745039X.2017.1390181 29058462

[B97] Nuamah E. Poaty Ditengou J. I. C. Hirwa F. Cheon I. Chae B. Choi N.-J. (2024). Dietary supplementation of tannins: effect on growth performance, serum antioxidant capacity, and immunoglobins of weaned piglets—a systematic review with meta-analysis. Antioxidants 13, 236. 10.3390/antiox13020236 38397834 PMC10886058

[B98] Ollagnier C. Mellino M.-R. Pradervand N. Tretola M. Dubois S. Durosoy S. (2025). Feed supplementation with potentiated zinc and/or tannin-rich extracts reduces ETEC infection severity and antimicrobial resistance genes in pig. Front. Vet. Sci. 12, 1494103. 10.3389/fvets.2025.1494103 40061905 PMC11887510

[B99] Orak H. Yagar H. Isbilir S. Demirci A. Gumus T. (2013). Antioxidant and antimicrobial activities of white, green and black tea extracts. Acta Aliment. 42, 379–389. 10.1556/AAlim.2013.2222

[B100] Óvilo C. Benítez R. Núñez Y. Peiró-Pastor R. García F. De Mercado E. (2025). Expression and structural analysis of taste receptor genes in Iberian and Duroc pigs. Genet. Sel. Evol. 57, 22. 10.1186/s12711-025-00968-0 40316903 PMC12046925

[B101] Pan L. Feng S. Li W. Zhu W. (2022a). Comparative digestion and fermentation characteristics of low-tannin or high-tannin sorghum grain in the porcine gastrointestinal tract. J. Anim. Sci. 100, skac300. 10.1093/jas/skac300 36075205 PMC9667962

[B102] Pan L. Li W. Gu X. M. Zhu W. Y. (2022b). Comparative ileal digestibility of gross energy and amino acids in low and high tannin sorghum fed to growing pigs. Anim. Feed Sci. Technol. 292, 115419. 10.1016/j.anifeedsci.2022.115419

[B103] Piwowarski J. P. Granica S. Kiss A. K. (2015). *Lythrum salicaria* L.—underestimated medicinal plant from European traditional medicine. A review. J. Ethnopharmacol. 170, 226–250. 10.1016/j.jep.2015.05.017 25985768

[B104] Puljula E. Walton G. Woodward M. J. Karonen M. (2020). Antimicrobial activities of ellagitannins against *Clostridiales perfringens, Escherichia coli, Lactobacillus plantarum* and *Staphylococcus aureus* . Molecules 25, 3714. 10.3390/molecules25163714 32824081 PMC7465317

[B105] Rajković E. Schwarz C. Tischler D. Schedle K. Reisinger N. Emsenhuber C. (2021). Potential of grape extract in comparison with therapeutic dosage of antibiotics in weaning piglets: effects on performance, digestibility and microbial metabolites of the ileum and colon. Animals (Basel) 11, 2771. 10.3390/ani11102771 34679793 PMC8532789

[B106] Rajković E. Schwarz C. Kapsamer S. B. Schedle K. Reisinger N. Emsenhuber C. (2022). Evaluation of a dietary grape extract on oxidative status, intestinal morphology, plasma acute-phase proteins and inflammation parameters of weaning piglets at various points of time. Antioxidants 11, 1428. 10.3390/antiox11081428 35892630 PMC9394324

[B107] Reggi S. Giromini C. Dell’Anno M. Baldi A. Rebucci R. Rossi L. (2020). *In vitro* digestion of chestnut and quebracho tannin extracts: antimicrobial effect, antioxidant capacity and cytomodulatory activity in swine Intestinal IPEC-J2 cells. Animals (Basel) 10, 195. 10.3390/ani10020195 31979207 PMC7070574

[B108] Reis de Souza T. C. Ávila Árres I. E. Ramírez Rodríguez E. Mariscal-Landín G. (2019). Effects of kafirins and tannins concentrations in sorghum on the ileal digestibility of amino acids and starch, and on the glucose and plasma urea nitrogen levels in growing pigs. Livest. Sci. 227, 29–36. 10.1016/j.livsci.2019.06.022

[B109] Ren Y.-Y. Zhang X.-R. Li T.-N. Zeng Y.-J. Wang J. Huang Q.-W. (2021). *Galla Chinensis*, a traditional Chinese medicine: comprehensive review of botany, traditional uses, chemical composition, pharmacology and toxicology. J. Ethnopharmacol. 278, 114247. 10.1016/j.jep.2021.114247 34052353

[B110] Rhouma M. Fairbrother J. M. Beaudry F. Letellier A. (2017). Post weaning diarrhea in pigs: risk factors and non-colistin-based control strategies. Acta Vet. Scand. 59, 31. 10.1186/s13028-017-0299-7 28526080 PMC5437690

[B111] Rodríguez-Hernández P. Reyes-Palomo C. Sanz-Fernández S. Rufino-Moya P. J. Zafra R. Martínez-Moreno F. J. (2023). Antiparasitic tannin-rich plants from the South of Europe for grazing livestock: a review. Animals (Basel) 13, 201. 10.3390/ani13020201 36670741 PMC9855007

[B112] Samtiya M. Aluko R. E. Dhewa T. (2020). Plant food anti-nutritional factors and their reduction strategies: an overview. Food Prod. Process. Nutr. 2, 6. 10.1186/s43014-020-0020-5

[B113] Santovito E. Greco D. Logrieco A. F. Avantaggiato G. (2018). Eubiotics for food security at farm level: yeast cell wall products and their antimicrobial potential against pathogenic bacteria. Foodborne Pathog. Dis. 15, 531–537. 10.1089/fpd.2018.2430 29874106

[B114] Schlittenlacher T. Knubben-Schweizer G. Dal Cero M. Vogl C. R. Maeschli A. Hamburger M. (2022). What can we learn from past and recent Bavarian knowledge for the future development of European veterinary herbal medicine? An ethnoveterinary study. J. Ethnopharmacol. 288, 114933. 10.1016/j.jep.2021.114933 34954268

[B115] Schneider L. I. Borba A. Medeiros J. M. D. Klein D. R. Poletti B. Rossi C. A. R. (2024). Black wattle (*Acacia mearnsii*) condensed tannin extract as feed additive in diets of weaned piglets. Cienc. Rural. 54, e20220515. 10.1590/0103-8478cr20220515

[B116] Shang Y.-F. Cao H. Ma Y.-L. Zhang C. Ma F. Wang C.-X. (2019). Effect of lactic acid bacteria fermentation on tannins removal in Xuan Mugua fruits. Food Chem. 274, 118–122. 10.1016/j.foodchem.2018.08.120 30372915

[B117] Sharma K. Kumar V. Kaur J. Tanwar B. Goyal A. Sharma R. (2021). Health effects, sources, utilization and safety of tannins: a critical review. Toxin Rev. 40, 432–444. 10.1080/15569543.2019.1662813

[B118] Singh M. N. Hemant K. S. Y. Ram M. Shivakumar H. G. (2010). Microencapsulation: a promising technique for controlled drug delivery. Res. Pharm. Sci. 5, 65–77. 21589795 PMC3093624

[B119] Smith A. H. Zoetendal E. Mackie R. I. (2005). Bacterial mechanisms to overcome inhibitory effects of dietary tannins. Microb. Ecol. 50, 197–205. 10.1007/s00248-004-0180-x 16222487

[B120] Smulikowska S. Pastuszewska B. Święch E. Ochtabińska A. Mieczkowska A. Nguyen V. C. (2001). Tannin content affects negatively nutritive value of pea for monogastrics. J. Anim. Feed Sci. 10, 511–523. 10.22358/jafs/68004/2001

[B121] Song Y. Luo Y. Yu B. He J. Zheng P. Mao X. (2021). Tannic acid extracted from gallnut prevents post-weaning diarrhea and improves intestinal health of weaned piglets. Anim. Nutr. 7, 1078–1086. 10.1016/j.aninu.2021.04.005 34738038 PMC8546364

[B122] Souza K. L. de Dias C. P. Callegari M. A. Friderichs A. Paes A. O. S. de Carvalho R. H. (2025). Performance and intestinal health of piglets in the nursery phase subjected to diets with condensed black wattle (*Acacia mearnsii*) tannin. Anim. Biosci. 38, 117–130. 10.5713/ab.24.0112 39210818 PMC11725734

[B123] Spreeuwenberg M. A. Verdonk J. M. Gaskins H. R. Verstegen M. W. (2001). Small intestine epithelial barrier function is compromised in pigs with low feed intake at weaning. J. Nutr. 131, 1520–1527. 10.1093/jn/131.5.1520 11340110

[B124] Steendam C. A. C. Tamminga S. Boer H. de Jong E.-J. Visser G. H. Verstegen M. W. A. (2004). Ileal endogenous nitrogen recovery is increased and its amino acid pattern is altered in pigs fed quebracho extract. J. Nutr. 134, 3076–3082. 10.1093/jn/134.11.3076 15514278

[B125] Sun J. Wang K. Xu B. Peng X. Chai B. Nong S. (2021). Use of hydrolyzed Chinese gallnut tannic acid in weaned piglets as an alternative to zinc oxide: overview on the gut microbiota. Anim. (Basel) 11, 2000. 10.3390/ani11072000 34359128 PMC8300422

[B126] Szabó C. Kachungwa Lugata J. Ortega A. D. S. V. (2023). Gut health and influencing factors in pigs. Anim. (Basel) 13, 1350. 10.3390/ani13081350 37106913 PMC10135089

[B127] Tang X. Xiong K. Zeng Y. Fang R. (2024). The mechanism of zinc oxide in alleviating diarrhea in piglets after weaning: a review from the perspective of intestinal barrier function. IJMS 25, 10040. 10.3390/ijms251810040 39337525 PMC11432186

[B128] Tong Z. He W. Fan X. Guo A. (2022). Biological function of plant tannin and its application in animal health. Front. Vet. Sci. 8, 803657. 10.3389/fvets.2021.803657 35083309 PMC8784788

[B129] Tretola M. Bee G. Silacci P. (2021). Gallic acid affects intestinal-epithelial-cell integrity and selected amino-acid uptake in porcine *in vitro* and *ex vivo* permeability models. Br. J. Nutr. 126, 492–500. 10.1017/S0007114520004328 33143768

[B130] Vadivel V. Biesalski H. K. (2012). Effect of certain indigenous processing methods on the bioactive compounds of ten different wild type legume grains. J. Food Sci. Technol. 49, 673–684. 10.1007/s13197-010-0223-x 24293686 PMC3550828

[B131] Van Parys A. Boyen F. Dewulf J. Haesebrouck F. Pasmans F. (2010). The use of tannins to control *Salmonella typhimurium* infections in pigs. Zoonoses Public Health 57, 423–428. 10.1111/j.1863-2378.2009.01242.x 19538452

[B132] Vargas-Magaña J. J. Aguilar-Caballero A. J. Torres-Acosta J. F. Sandoval-Castro C. A. Hoste H. Capetillo-Leal C. M. (2013). Tropical tannin-rich fodder intake modifies saliva-binding capacity in growing sheep. Animal 7, 1921–1924. 10.1017/S1751731113001651 24093808

[B133] Verhelst R. Schroyen M. Buys N. Niewold T. (2010). The effects of plant polyphenols on enterotoxigenic *Escherichia coli* adhesion and toxin binding. Livest. Sci. 133, 101–103. 10.1016/j.livsci.2010.06.035

[B134] Verhelst R. Schroyen M. Buys N. Niewold T. A. (2013). *E. coli* heat labile toxin (LT) inactivation by specific polyphenols is aggregation dependent. Vet. Microbiol. 163, 319–324. 10.1016/j.vetmic.2012.12.039 23391440

[B135] Verhelst R. Schroyen M. Buys N. Niewold T. (2014). Dietary polyphenols reduce diarrhea in enterotoxigenic *Escherichia coli* (ETEC) infected post-weaning piglets. Livest. Sci. 160, 138–140. 10.1016/j.livsci.2013.11.026

[B136] Vitti D. M. S. S. Nozella E. F. Abdalla A. L. Bueno I. C. S. Filho J. C. S. Costa C. (2005). The effect of drying and urea treatment on nutritional and anti-nutritional components of browses collected during wet and dry seasons. Anim. Feed Sci. Technol. 122, 123–133. 10.1016/j.anifeedsci.2005.04.007

[B137] Waghorn G. C. Ulyatt M. J. John A. Fisher M. T. (1987). The effect of condensed tannins on the site of digestion of amino acids and other nutrients in sheep fed on *Lotus corniculatus* L. Br. J. Nutr. 57, 115–126. 10.1079/BJN19870015 3801377

[B138] Wang Y. Jin L. Ominski K. H. He M. Xu Z. Krause D. O. (2013). Screening of condensed tannins from Canadian prairie forages for anti–*Escherichia coli* O157:H7 with an emphasis on purple prairie clover (*Dalea purpurea* Vent). J. Food Prot. 76, 560–567. 10.4315/0362-028X.JFP-12-259 23575115

[B139] Wang J. Xiao H. Zhu Y. Liu S. Yuan Z. Wu J. (2019). Tannic acid induces the mitochondrial pathway of apoptosis and S phase arrest in porcine intestinal IPEC-J2 cells. Toxins (Basel) 11, 397. 10.3390/toxins11070397 31323908 PMC6669611

[B140] Wang M. Huang H. Hu Y. Huang J. Yang H. Wang L. (2020). Effects of dietary microencapsulated tannic acid supplementation on the growth performance, intestinal morphology, and intestinal microbiota in weaning piglets. J. Anim. Sci. 98, skaa112. 10.1093/jas/skaa112 32255185 PMC7199885

[B141] Wang M. Huang H. Wang L. Yin L. Yang H. Chen C. (2022). Tannic acid attenuates intestinal oxidative damage by improving antioxidant capacity and intestinal barrier in weaned piglets and IPEC-J2 cells. Front. Nutr. 9, 1012207. 10.3389/fnut.2022.1012207 36407512 PMC9672516

[B142] Wei X. Tsai T. Howe S. Zhao J. (2021). Weaning induced gut dysfunction and nutritional interventions in nursery pigs: a partial review. Animals (Basel) 11, 1279. 10.3390/ani11051279 33946901 PMC8146462

[B143] Widsten P. Cruz C. D. Fletcher G. C. Pajak M. A. McGhie T. K. (2014). Tannins and extracts of fruit byproducts: antibacterial activity against foodborne bacteria and antioxidant capacity. J. Agric. Food Chem. 62, 11146–11156. 10.1021/jf503819t 25339414

[B144] Williams A. R. Fryganas C. Ramsay A. Mueller-Harvey I. Thamsborg S. M. (2014a). Direct anthelmintic effects of condensed tannins from diverse plant sources against *Ascaris suum* . PLoS One 9, e97053. 10.1371/journal.pone.0097053 24810761 PMC4014605

[B145] Williams A. R. Ropiak H. M. Fryganas C. Desrues O. Mueller-Harvey I. Thamsborg S. M. (2014b). Assessment of the anthelmintic activity of medicinal plant extracts and purified condensed tannins against free-living and parasitic stages of *Oesophagostomum dentatum* . Parasit. Vectors 7, 518. 10.1186/s13071-014-0518-2 25406417 PMC4240858

[B146] Williams A. R. Krych L. Fauzan Ahmad H. Nejsum P. Skovgaard K. Nielsen D. S. (2017). A polyphenol-enriched diet and *Ascaris suum* infection modulate mucosal immune responses and gut microbiota composition in pigs. PLoS One 12, e0186546. 10.1371/journal.pone.0186546 29028844 PMC5640243

[B147] Xiong X. Tan B. Song M. Ji P. Kim K. Yin Y. (2019). Nutritional intervention for the intestinal development and health of weaned pigs. Front. Vet. Sci. 6, 46. 10.3389/fvets.2019.00046 30847348 PMC6393345

[B148] Xu T. Ma X. Zhou X. Qian M. Yang Z. Cao P. (2022). Coated tannin supplementation improves growth performance, nutrients digestibility, and intestinal function in weaned piglets. J. Anim. Sci. 100, skac088. 10.1093/jas/skac088 35298652 PMC9109020

[B149] Yi H. Wang Z. Yang B. Yang X. Gao K. Xiong Y. (2023). Effects of zinc oxide and condensed tannins on the growth performance and intestinal health of weaned piglets in ETEC-challenged environment. Front. Microbiol. 14, 1181519. 10.3389/fmicb.2023.1181519 37180229 PMC10172512

[B150] Yu J. Song Y. Yu B. He J. Zheng P. Mao X. (2020). Tannic acid prevents post-weaning diarrhea by improving intestinal barrier integrity and function in weaned piglets. J. Anim. Sci. Biotechnol. 11, 87. 10.1186/s40104-020-00496-5 32884745 PMC7460753

[B151] Zhang Q. Zhang L. Du L. Zhang Y. Yi D. Zhao D. (2023). Dietary supplementation of natural tannin relieved intestinal injury and oxidative stress in piglets challenged with enterotoxigenic *Escherichia coli* . Czech J. Anim. Sci. 68, 296–305. 10.17221/148/2022-CJAS

