# Supplementary material for: The eubiotic perspective on utilization of tannins in phytotherapy and nutrition of pigs
Source: Front Pharmacol. 2025 Aug 25;16:1649388. doi: 10.3389/fphar.2025.1649388 (PMC12415002; doi:10.3389/fphar.2025.1649388)
Supplement: Supplementary file 1 [file Table1.pdf]

Table S1. Impact of tannins on pig production

	Source	Feed supplement (Form)	Tannin content	Type of tannin*	Test model	Effects on observation*	Dose of feed supplement*	Duration	Ref.
1	Kenwood	-	-	CT	72 weaned piglets (Duroc x Landrace x Yorkshire), 26 d-old, average BW: 8.40 kg<br>ETEC-K88-challenged environment; bacterial solution (6 x 10^8 CFU/ml) was sprayed on day 1, 5, 9, 13, 17, 21, and 25 of the trial	BW, ADG, ADFI and F/G =<br>Reducing rate and index of diarrhea ↑ (d 0-14, 15-28 and 0-28)<br>Chao1, Shannon and Simpson = in colon digesta<br>Microbial composition = in colon digesta	Con: basal diet<br>ZnO: basal diet + 1.5g/kg zinc oxide<br>CT: basal diet + 1 g/kg condensed tannins<br>ZnO + CT: basal diet + 1.5 g/kg ZnO + 1 g/ kg condensed tannins	28 d	(Yi et al. 2023)
2	Chestnut and Quebracho	Silvafeed® Nutri P, Silvateam Italy	(75 % tannins)	CT and HT	88 weaned piglets (Swiss Large White), 26 d-old, average BW: 7.8 kg<br>Infection of the piglets on d 4 with a solution of 10^{10} ETEC F4	BW, ADG and ADFI and feed efficiency =<br>Duration of diarrhea and daily prevalence of diarrhea =<br>ETEC F4 excretion =	Con: basal diet:<br>TAN + pZnO-150: basal diet + 7.5 g/kg tannin extract (quebracho and chestnut) + 015 g Zn/kg pZnO<br>pZnO: basal diet + 0.15 g pZnO<br>TAN: basal diet + 7.5 g/kg tannin extract (quebracho and chestnut)	13 (± 1); nine days (± 1) after infection	(Ollagnier et al., 2025)
3	Chestnut (*Castanea sativa* Mill)	Chestnut meal (ground)	-	HT	16 pigs (Landrace x Yorkshire x Duroc); average BW: 95 ± 2 kg<br>Housed in metabolic cages	Quadratic effect on tannin and DM intake with higher chestnut supplementation in the diet, CP, EE and crude ash intake =<br>Highest intake of DM and tannin ↑ in the T10 group<br>DM, CP, EE, CA and tannin digestibility ↓ with higher tannin supplementation	Con: basal diet (0.16 % tannins per DM)<br>T 5: basal diet + 5 % chestnut meal (0.17 % tannins per DM)<br>T 10: basal diet + 10 % chestnut meal (0.18 % tannins per DM)<br>T 15: basal diet + 15 % chestnut meal (0.19 % tannins per DM)	14 d adaptation period, 7 d collection period	(Lee et al., 2016)

4	Chestnut (*Castanea sativa* Mill)	Coated tannins (hydrogenated palm oil was used as coating material)	25 %	HT	180 weaned barrows (Duroc x Landrace x Yorkshire), 28 d-old, average BW: 8.6 kg	ADG ↑, F/G ↓, ADFI =<br>CP digestibility ↑; CA, crude fat, Ca and P =<br>Diarrhea incidence ↓<br>Chao1, Observed species, Shannon and Simpson indices =<br>*Lachnospiraceae* was the family with the highest abundant in the Con group, while *Ruminococcaceae* was the one with the highest abundant in the Tan group ↑<br>rel. abundance of *Faecalitalea*, *Acidaminococcus*, *Methanobrevibacter smithii*, *Faecalibacterium* and *Turicibacter* ↑	Con: basal diet (include $ZnSO_4$)<br>Tan: basal diet + 1,500 mg/kg coated tannins<br>Add. ZnO: basal diet + 1,600 mg/kg as ZnO	20 d	(Xu et al., 2022)
5	Chestnut wood extract (*Castanea sativa* Mill)	Farmatan®	75 % tannins	HT	48 weaned piglets (German Landrace x Pietrain), 28 d-old, average BW: 8.23 ± 0.93 kg<br>housed in metabolic cages 4 d adaption period (all same diet)	number of viable lactobacilli ↑ (tended) in jejunum digesta<br>number of coliforms ↑ (tended) in caecum digesta<br>reduced ammonia concentration ↓ (tended) in caecum digesta<br>propionic acid ↓ (tended) in caecum digesta in 1.13 and 4.5 g/kg group in relation to 2.25 g/kg and Con<br>iso-butyric and iso-valeric acid ↓ in caecum digesta<br>*n*-butyric acid and total volatile fatty acids ↓ in 1.13 g/kg and 4.5 g/kg than in the others	Con: basal diet<br>1.13 g/kg: basal diet + 1.13 g/kg tannins<br>2.25 g/kg: basal diet + 2.25 g/kg tannins<br>4.5 g/kg: basal diet + 4.5 g/kg tannins<br>Corn was replaced by the tannin product	28 d	(Biagi et al., 2010)
6	Chestnut wood extract (*Castanea sativa* Mill)	Tanin Sevnica d.d., Slovenia	-	HT	504 pigs (Swedish Landrace x Large White), 23 d-old piglets	No diarrhea =	Con: basal diet<br>OA: basal diet + 0.35 % organic acids (FraAcidDry)<br>OA + T: 0.35 % extract (0.19 % tannins) + 0.16 % of organic acids (five different)	104 d	(Brus et al., 2013)

7	Quebracho tannin extract (*Schinopsis lorentzii*) (heartwood)	MGM-P	> 50 %	CT	36 weaner piglets (Duroc x Landrace x Yorkshire), 21-d-old piglets	BW, ADG, ADFI and FCR =; no diarrhea =, only some mushed feces in the HD group	Con: basal diet<br>LD: basal diet + 5 g/kg MGM-P<br>HD: basal diet + 10 g/kg MGM-P<br>p. Con (positive control): basal diet + 0.1 g/kg flavomycin	21 d	(Ma et al., 2024)
8	Quebracho tannin extract (*Schinopsis* spp.)	MGM-P	> 50 %	CT	30 weaner piglets (Duroc x Landrace x Yorkshire), 21-d-old piglets	No diarrhea in 0.3 % MGM-P group, diarrhea incidence in Con and 0.2 % MGM-P ↓ of 12.5 %	Con: basal diet<br>0.2 % MGM-P: basal diet + 2 g/kg MGM-P<br>0.3 % MGM-P: basal diet + 3 g/kg MGM-P	20 d	(Ma et al., 2021)
9	Quebracho tree tannin extract and Chestnut tree tannin extract (*Schinopsis* spp., *Castanea* spp.)	Silvafeed Nutri P/ENC for Swine	75 g tannins/100 g DM	CT and HT	120 weaned piglets (Large White x Landrace), 28-d-old piglets	Diarrhea occurrence:<br>0-14 d: 3.39 % in Con and 5.00 % in Ch/Qu<br>14-28 d: 18.64 % in Con and 15.52 % in Ch/Qu<br>28-40 d: 1.69 % in Con and 3.45 % in Ch/Qu<br>Principle coordinate analysis did not result in a different microbiota of the piglets feces, while PERMANOVA show a separation between the groups in terms of the microbiome<br>Chao1 = in feces<br>Observed species, Shannon and Simpson indices ↑ in feces<br>Rel. abundance of *Bacteroidetes* and *Actinobacteria* ↓, *Spirochaetae* and *Cyanobacteria* in feces ↑<br>Rel. abundance of *Clostridiaceae* as well as *Peptococcaceae Spirochaetaceae* as well as *Peptostreptococcaceae* ↑, rel. abundance of *Prevotellaceae*, *Eubacteriaceae, Coriobacteriaceae, Desulfovibrionaceae, Veillonellaceae, Rikenellaceae as well as Deferribacteraceae,* ↓<br>Rel. abundance of *Shuttleworthia*, *Pseudobutyrivibro*, *Anaerostipes*, *Solobacterium* and *Peptococcus* ↑, rel. abundance of *Synotrophococcus*, *Mitsuokella*, *Sharpea*, *Atopbium* and *Prevotella* ↓<br>Butyrate in feces ↑, valerate in feces ↓	Con: basal diet<br>Ch/Qu: basal diet + 1.25 % extract (quebracho tree tannin extract and chestnut tree tannin extract)	One week of adaptation (all basal diet) + 40 d treatment diets	(Caprarulo et al., 2020; Miragoli et al., 2021)

10	Quebracho extract (*Schinopsis* spp.)	Unitan Sacia, Quebracho Superior ATO	120 g/kg catechin equivalent, 650 g/kg total phenols, 9 g/kg CP	CT	12 weaned barrows (Dutch Landrace x Finnish Landrace) x Great Yorkshire) average BW at arrival: 9 kg ± 0.6 kg<br>Housed in metabolic cages, T-caecum cannula<br>[^{15} N] -*L*-leucine one group got the isotope orally and one group via infusion into the jugular vein<br>Diets were fed in a crossover design	^{15}N -enrichment in the digesta ↑, dilution factor ↑, N AID ↓, Calculated endogenous nitrogen recovery (g/d or g/kg DMI) of approximately 300 % ↑, True N digestibility =<br>Endogenous nitrogen recovery with both methods ↑True N digestibility with peptide alimental ultrafiltration method ↓, True N digestibility values based on the ^{15}N-isotope dilution technique =, N AID ↓<br>Different amino acid pattern of endogenous protein due to the Tan diet, proline, arginine, glycine and leucine, tyrosine, methionine, phenylalanine as well as alanine ↑, threonine, glutamic acid, serine and isoleucine ↓	Con: cornstarch, and enzyme-hydrolyzed casein<br>Tan: con diet + 4 % quebracho extract	28 days of adaptation period<br>10 d period<br>got the diet from the farm for the first 6 days	(Steendam et al., 2004a)<br>(Steendam et al., 2004b)
11	Black wattle (*Acacia mearnsii*)	74 % black wattle condensed tannins extract + 26 % soy lecithin	74 % condensed tannins	CT	88 weaned piglets (crossbred); BW: 8.02 ± 1.21 kg	BW, ADFI, DWG, FCR =; pre-starter I (0-7): liquid and pasty feces incidence ↓; Pre-starter II (8-22): incidence of liquid (tendency) and pasty feces ↓	Con sim: simple diet (lower concentration of dairy products, no ZnO, no acidifier and no yeast)<br>SD + T: simple diet + 1,850 mg/kg tannins (2.5 g/kg commercial product)<br>Con com: complex diet (higher content of dairy products, ZnO, acidifier, yeast)<br>CD + T: complex diet + 1,850 mg/kg tannins (2.5 g/kg commercial product)	28 d (0-7 d pre-starter I, 8-21 d pre-starter II, 22-28 d starter)	(Schneider et al., 2024)
12	Chestnut wood extract (*Castanea sativa* Mill)	Saviotan Feed	82 % tannins, 2.5 % CP	HT	18 pigs (hybrid); average BW 76 kg	Dry matter and nitrogen digestibility in 0.5 % group ↓ in relation to Con group (0.25 % group was intermediate), nitrogen retained and protein biological value =	Con: basal diet<br>Tan 0.25 %: basal diet + 0.5 % extra CP + 250 g tannin/100 kg feed<br>Tan 0.5 %: basal diet + 1 % extra CP + 500 g tannins/ 100 kg feed	40 d<br>After 20 d one week in a metabolic cage	(Antongiovanni et al., 2007)
13	Chestnut wood extracts (*Castanea sativa* Mill)	SavitanFeed (Gruppo Mauro Saviola Srl, Radicofani, Siena, Italy)	75 % tannins	HT	18 barrows (Italian Landrace x Italian Large White); average BW during the test period: 153 ± 4 kg	DM, OM, CP, EE, aNDFom, ADFom ash and GE digestibility =, Daily fecal N excretion =, Urine N extretion =	Con: basal diet<br>LP: low protein diet (5 % less soybean meal)<br>LPT: low protein diet + tannin 5.3 g/kg tannin extract (4 g/kg tannic acid equivalent)<br>Restricted feeding during the trial	3 periods, each period 14 d: 7 d adaptation period. 7 d collection period (feces and urine)	(Galassi et al., 2019)

14	Gallnut (*Galla chinensis*)	Tannic acid (TA)	-	HT	432 weaned piglets, average BW: 7.05 ± 1.05 kg	BW, ADFI, ADG and F/G =<br>Rel abundance of *Proteobacteria* were higher and *Firmicutes* in 1.5 g/kg TA ↓ compared to Con<br>Shannon index =<br>OTUs ↓ due to the inclusion of 3 g/kg TA<br>Rel abundance of *Candidatus Brocadia*, *[Eubacterium] oxidoreducens group* and *Escherichia-Shigella* ↓ in 3.0 g/kg TA than in Con	Con: basal diet<br>1.5 g/kg TA: basal diet + 1.5 g/kg TA<br>3.0 g/kg TA: basal diet + 3.0 g/kg TA<br>1.8 g/kg ZnO: basal diet + 1.8 g/kg ZnO	21 d	(Deng et al., 2024)
15	Gallnut (*Galla chinensis*)	Tannalbin (Guangzhou Insighter Biotechnology Co. Ltd., Guangzhou China)	51 % tannic acid and 40.17 % protein	HT	180 weaned piglets (Duroc x Landrace x Yorkshire) 21 d-old; average BW: 7.77 ± 0.17 kg<br>5 d adaptation period	BW, ADG, ADFI and FCR =<br>nutrient digestibility = in the groups up to 0.2% TA<br>digestibility of ether extract ↑ in 0.4% TA, digestibility of CP and gross energy ↓ in 0.4% TA relation to Con, 0.05, 0.1% of TA, respectively Con and 0.05%<br>CP, gross energy, crude fiber and dry matter digestibility (linear) ↓ with higher TA in the diet<br>0.2 and 0.4% TA diarrhea ↓, incidence of diarrhea ↓ (linear) with higher TA in diet<br>*Bacillus* number in caecum digesta ↑ TA (dose dependent), *Bacillus* counts ↑ in colon digesta of 0.4 % TA in relation to Con, 0.1 and 0.2 % TA<br>*E. coli*. in colon digesta ↓ of 0.1 % TA piglets in comparison to Con and 0.4 % TA as well as reduced value ↓ in 0.4 % TA in relation to 0.05 % TA<br>Cecum digesta: Butyric acid ↑ in all TA groups, isovaleric acid ↑ in 0.1 %, 0.2 % and 0.4 % group than in Con, propionic acid ↑ in 0.2 % TA than in 0.1, 0.05 % TA and Con, and 0.4 % TA ↑ than the Con<br>Colon digesta: Acetic acid ↑ in 0.4 % TA than in the others, isobutyric acid ↑ in 0.05 % TA in relation to 0.2 and 0.4 % TA and 0.4 % TA ↓ than 0.1 % TA	Con: basal diet<br>0.05% TA: basal diet + 0.05% tannalbin<br>0.1% TA: basal diet + 0.1% tannalbin<br>0.2% TA: basal diet + 0.2% tannalbin<br>0.4% TA: basal diet + 0.4% tannalbin	29 d	(Song et al., 2021)

16	Black wattle (*Acacia mearnsii*)	Tanfeed	Approximately 47.8 % tannins,	CT	200 weaned piglets (PIC), 22 d-old piglets, average BW: 6.0 ± 0.9 kg	ADG ↑ (tended) at the end of the trial, ADFI, ADG, BW and G:F day 29-43 ↑, number of diarrhea cases ↓, ATTD of DM, CP, ash or gross energy =, Chao1 and Shannon indices = in rectum content<br>Rel. abundance of *Brevibacillus* spp. in rectum ↑, *Enterococcus* Spp. abundance in rectum =	Con: basal diet<br>ENR + ZnO: basal diet + 10 mg/kg of enramycin + 2,500 mg/kg of zinc oxide during 21 days<br>BUT: basal diet + 900 mg/kg of sodium butyrate<br>TAN: basal diet + 2,000 mg/kg of condensed tannin	42 d<br>4 feeding phases	(Souza et al., 2025)
17	Natural tannins	Wuhan Yuancheng Gongchuang Technology Ltd (Shanghai, China)	-	-	32 piglets (Duroc x Landrace x Yorkshire), 7 d-old<br>ETEC strain K 88, oral inoculation: 5 x 10^9 CFU	ADG ↑ in Tan + ETEC group compared to ETEC group, while Con and ZnO + ETEC were significantly higher than the two other groups during d 8 and 9<br>ADFI ↓ during d 1-7 in ZnO + ETEC in relation to the others<br>ADFI ↓ during d 8-9 in the groups Tan + ETEC and ZnO + ETEC than in the Con and ETEC group; incidence diarrhea ↑ in ETEC groups, diarrhea rate ↓ in Tan	Con: basal diet (milk substitute) without challenge<br>ETEC group: basal diet + challenge with ETEC<br>Tan + ETEC: basal diet + 2 g/kg Tan + challenged with ETEC<br>ZnO + ETEC: basal diet + 3 g/kg ZnO + challenge with ETEC	10 d<br>3 d adaptation period (only milk substitute), 7 d with supplements	(Zhang et al., 2023)
18	ALSOK<br>Cocoa beans (*Theobroma cacao*)<br>Grape seed (*Vitis vinifera* L.)	ALOSK: hydrolysable tannin extract (medium and highmolecular weight polyphenols<br>Omnicoa: coca bean extract (proanthocyanindin, low molecular weight oligomers)<br>Omnivin: grape seed extract (poranthocyanidin, low molecular weight oligomers)	-	HT and CT	40 weaned piglets, 3 weeks old; half of the group in each treatment was infected on d 6 and 7 with 5 ml ETEC suspension of 10^9CFU/ml	FCR and ADFI =, ADG ↓ from d 6 to 13 in Omnicoa group	Con: basal diet<br>ALOSK: basal diet + 1 % ALOSK extract<br>Omnicoa: basal diet + 1 % Omnicoa extract<br>Omnivin: basal diet + 1 % Omivin extract	16 d	(Verhelst et al., 2014)

19	Extract of dried grapes (*Vitis vinifera* L.)	Water-based grape extract (Biomin Holding GmbH, Getzersdorf, Austria)	> 40% total polyphenols, > 30% procyanidins, < 5% water	CT	180 weaned piglets (DanBred x Piétrain) 23 d-old piglets; average BW: 6.9 ± 0.1 kg	ATTD of ash and acidic-hydrolyzed EE ↑<br>ATTD of DM, OM, gross energy, CP and P ↑ over whole trial period<br>Microbial metabolites in ileum digesta =, Lactic acid in colon digesta ↑ (tendency)after 27/28 days, ammonia content in ileum digesta ↓ (tendency) in relation to Con after 27/28 days	Con: basal diet<br>PC: basal diet + amoxicillin for the first 5 days of the trial<br>GE: basal diet + 150 g/t grape extract	56 d<br>starter period: 1-13 d<br>grower period: 14-56	(Rajković et al., 2021)
20	Grape tannins (*Vitis* spp.)	Grape tannins (Prescott & Co., Mississauga, Ontario, Canada)	-	-	48 weaned barrows (Duroc x Yorkshire), 21-d old; average BW by start of the treatment diets: 9.42 ± 0.14 kg<br>T-cannula in the ileum, metabolic cage	AID of Threonine and Isoleucine ↓	12 d of basal diet + 3 d with a casein diet<br>7 diets among others<br>Con: basal diet<br>Tan: 15 g/kg tannins		(Myrie et al., 2008)
21	Grape seed extract (*Vitis* spp.)	Tianjin Jianfeng Natural Product R&D Co., Ltd (Tianjin China)	79.28 % oligomeric procyanidins	CT	120 weaned piglets (Duroc x Landrace x Yorkshire), 21 d-old piglets, average BW: 6.52 kg ± 0.18 kg	d 14:<br>AID of gross energy ↓ in T150 in comparison with Con and T50, AID of CP ↓ in the T150 and T100 group in relation to the other two, ATTD of gross energy ↓ in T150 group than in Con and T50, ATTD of CP ↓ in the T150 group compared to the other groups, ATTD of CP ↓ in the T100 group than to Con group<br>d 28:<br>AID of DM and EE ↓ in group T100 and T150 compared to Con and T50, AID of CP ↓ in T150 group in comparison to T50 and Con, AID of gross energy and ATTD of DM and CP ↓ in the T150 group in relation to the other groups	Con: basal diet<br>T50: basal diet + 50 mg/kg grape seed extract<br>T100: basal diet + 100 mg/kg grape seed extract<br>T150: basal diet + 150 mg/kg grape seed extract	28 d	(Li et al., 2020)
22	Dehydrated Sainfoin (*Onobrychis viciifolia*) (*Perly* cultivar)	-	Estimated content of condensed tannins in the four treatment diets: 4.0, 8.0 and 12.0 g/kg DM	CT	48 growing pigs (Swiss Large White EM), average BW 24.8 ± 5.1 kg	BW, ADG, ADFI, G:F over the whole trial period =	Con: basal diet<br>T5: basal diet + 5 % sainfoin<br>T10: basal diet + 10 % sainfoin<br>T15: basal diet + 15 % sainfoin	Grower diet: 25-60 kg BW (48 d)<br>Finisher diet: 60-105 kg BW (52 d)<br>End of the trial: 172 ± 3.9 d	(Seoni et al., 2021)

23	*Ceratonia siliqua* L.	Carob pulp	Diet without tannins had 3.6 g carob pulp internal total CT-eq and the diet with tannins had 19.0 g carob pulp internal total CT-eq/kg	Mainly CT	220 pigs ((Landrace x Large White) x Danbred Duroc) 130 d-old pigs; average BW 78 ± 8.5 kg	130-151 d, 4 % higher FCR ↑ with 20 % carob pulp (C + D), BW, ADG and ADFI =, growth performance over the whole trial =, CP coefficient of ATTD ↓, ether extract and hemicellulose coefficient of ATTD ↑ in 20 % carob pulp (C + D), Fecal DM =	A: with 30 IU/kg vitamin E, without carob pulp<br>B: diet with 300 IU/kg vitamin E, without carob pulp<br>C: diet with 30 IU/kg vitamin E, with 20 % carob pulp<br>D: diet with 300 IU/kg vitamin E and 20 % carob pulp	40 d	(Bottegal et al., 2024)
24	Sorghum (*Sorghum* spp.)	Low or high tannin sorghum	Low tannin sorghum: 3.7 mg/g CE<br>High tannin sorghum: 54.2 mg/g CE<br>CE: (+)-catechin equivalents	CT	12 barrows (Duroc x (Landrace x Yorkshire)); average BW: 25.5 ± 2.5 kg<br>Pigs had a T-cannula at the distal ileum<br>Metabolic cages	AID ↓ and ATTD of DM, CP and gross energy ↓ in the HTS group compared to LTS<br>Hind gut digestibility =	LTS: diet with 96.6 % low tannin sorghum<br>HTS: diet with 96.6 % high tannin sorghum	10 d<br>5 d adaptation period<br>3 d fecal collection<br>2 d ileum digesta collection	(Pan et al., 2022a)
25	Sorghum (*Sorghum* spp.)	-	-	-	24 castrated pigs (Landrace x Duroc); average BW: 60 ± 5 kg<br>Pigs had a T-cannula at the terminal ileum	AID of DM ↑ in T 1.4 than in the other groups, AID of CP ↑ in T 1.4 in relation to T 4.6, AID of the most amino acids ↑ in the T1.4 group, while T 4.6 often had the lowest values except for proline<br>AID of proline ↓ with higher tannin content in the diet	T 1.4: diet that had 1.4 g/kg tannins in the diet<br>T 4.6: diet that had 4.6 g/kg tannins in the diet<br>T 9.8: diet that had 9.8 g/kg tannins in the diet<br>T10.0: diet that had 10.0 g/kg tannins in the diet	5 d adaptation period, 2 d collection of ileal digesta	(Mariscal-Landín et al., 2004)

26	Sorghum (*Sorghum* spp.)	-	-	-	32 castrated pigs (Landrace x Duroc); average BW: 53 ± 7 kg<br>Pigs had a T-cannula at the terminal ileum	DM and CP AID =<br>High content of tannins resulted in a decreased AID of arginine, glycine and proline ↓<br>Proline AID was negative or very low in the diets with tow diets with the highest tannin content	Eight different Sorghum varieties were tested from two different suppliers:<br>8172: diet with 57.1 g/kg tannins<br>8428: diet with 47.2 g/kg tannins<br>8443: diet with 4.7 g/kg tannins<br>8641: diet with 6.6 g/kg tannins<br>D-45: diet with 5.3 g/kg tannins<br>D-65: diet with 12.2 g/kg tannins<br>D-68: diet with 8.9 g/kg tannins<br>D-69: diet with 0.2 g/kg tannins		(Mariscal-Landín et al., 2004)

27	Sorghum grain (*Sorghum* spp.)	-	-	CT	18 barrows (Duroc x (Landrace x Yorkshire)); average BW: 27.6 ± 3.5 kg<br><br>Pigs had a T-cannula at the distal ileum<br><br>Trial design: 9 x 3 Yourden square design	AID, hind gut digestibility and ATTD of CP and GE ↑ in the LTS<br><br>SID of lysine, threonine, valine, histidine, arginine, serine, glutamic acid as well as aspartic acid ↓ in the HTS groups<br><br>Negative correlation of tannin and total phenolic content in the grain in relation to AID, ATTD and hindgut digestibility of GE as well as CP and SID of lysine, threonine, histidine, valine as well as arginine	Diet without nitrogen, to correct the basal endogenous nitrogen losses<br><br>Treatment diets with 966 g/kg sorghum in the diet<br><br>Low tannin sorghum (LTS):<br><br>T 2.4: diet with a sorghum grain that resulted in a condensed tannin level of 2.4 g/kg<br><br>T 3.3: diet with a sorghum grain that resulted in a condensed tannin level of 3.3 g/kg<br><br>T 0.5: diet with a sorghum grain that resulted in a condensed tannin level of 0.5 g/kg<br><br>T 4.2: diet with a sorghum grain that resulted in a condensed tannin level of 4.2 g/kg<br><br>High tannin sorghum (HTS):<br><br>T 15.9: diet with a sorghum grain that resulted in a condensed tannin level of 15.9 g/kg<br><br>T 32.0: diet with a sorghum grain that resulted in a condensed tannin level of 32.0 g/kg<br><br>T 47.6 diet with a sorghum grain that resulted in a condensed tannin level of 47.6 g/kg<br><br>Restricted feeding during the trial	3 periods, 10 d each: 5 d adaptation, 3 d fecal collection, 2 d ileal digesta collection	(Pan et al., 2022b)

***General abbreviations and terms:** CT – Condensed tannins; HT – Hydrolysable tannins; Tan – Tannin supplemented diet; Con – Control group; d – day; $ZnSO_4$ – Zinc sulfate; ZnO – Zinc oxide; pZnO – Potentiated form of ZnO; OA – Organic acids; ETEC – Enterotoxigenic *Escherichia coli* (pathogenic *E. coli* strain used for challenge); HD – High tannin or high dose group; LD – Low tannin or low dose group; LTS – Low tannin sorghum; HTS – High tannin sorghum TA – Tannic acid; PC – Positive control (e.g., with antibiotics); NC – Negative control (e.g., without additives); MGM-P – commercial quebracho tannin extract; LP – Low protein diet; CD – Complex diet with tannin; SD – Simple diet; Ch/Qu – Chestnut and quebracho tannin blend; ALOSK / Omnicoa / Omivin – Commercial feed additive or plant extract products; CE – (+)-catechin equivalents; **Performance Parameters:** BW – Body weight; ADG – Average daily gain; ADFI – Average daily feed intake; FCR – Feed conversion ratio; DWG – Daily weight gain; G:F – Gain to feed ratio; F/G – Ratio of feed to gain; **Digestibility and Intake Parameters:** DM – Dry matter; CP – Crude protein; EE – Ether extract (fat); CA – Crude ash; OM – Organic matter; GE – Gross energy; aNDFom – Amylase-treated neutral detergent fiber, organic matter basis; ADFom – Acid detergent fiber, organic matter basis; ATTD – Apparent total tract digestibility; AID – Apparent ileal digestibility; SID – Standardized ileal digestibility; N AID – Nitrogen apparent ileal digestibility; **Nitrogen and Protein Utilization:** N retention – Nitrogen retained in body; N excretion – Nitrogen lost via feces or urine; BV – Biological value of protein; ^{15}N-enrichment – Isotopic labeling to track nitrogen metabolism; Endogenous N recovery – Measurement of internally secreted nitrogen in the digestive tract; Dilution factor – From ^{15}N isotope studies, reflects endogenous protein loss; **Microbiota and Metabolites:** OTUs – Operational taxonomic units (microbial diversity count); VFA – Volatile fatty acids; SCFA – Short-chain fatty acids; Bacteroidetes / Firmicutes / Proteobacteria / Actinobacteria – Major bacterial phyla; Butyrate / Propionate / Acetate / Valerate / Iso-butyric / Iso-valeric acid – Key microbial fermentation products; Lactic acid – Product of

lactobacilli fermentation; Ammonia – Byproduct of protein fermentation; **Statistical/Biodiversity Indices:** Shannon index – Measure of microbial diversity (richness and evenness); Simpson index – Another diversity measure, emphasizing dominant species; Chao1 index – Estimator of species richness; PERMANOVA – Permutational multivariate analysis of variance; PCoA – Principal coordinate analysis.

References

Antongiovanni, M., Minieri, S., and Petacchi, F. (2007). Effect of tannin supplementation on nitrogen digestibility and retention in growing pigs. *Ital. J. Anim. Sci.* 6, 245–247. doi: 10.4081/ijas.2007.1s.245

Biagi, G., Cipollini, I., Paulicks, B. R., and Roth, F. X. (2010). Effect of tannins on growth performance and intestinal ecosystem in weaned piglets. *Arch. Anim. Nutr.* 64, 121–135. doi: 10.1080/17450390903461584

Bottegal, D. N., Latorre, M. Á., Lobón, S., Verdú, M., and Álvarez-Rodríguez, J. (2024). Fattening pigs with tannin-rich source (*Ceratonia siliqua* L.) and high doses of vitamin E: effects on growth performance, economics, digestibility, physiology, and behaviour. *Animals* 14, 1855. doi: 10.3390/ani14131855

Brus, M., DolinŠek, J., DolinŠeK, J., and CenCiČ, A. (2013). Effect of chestnut (*Castanea sativa* Mill.) wood tannins and organic acids on growth performance and faecal microbiota of pigs from 23 to 127 days of age. *Bulg. J. Agric. Sci.*, 841–847.

Caprarulo, V., Hejna, M., Giromini, C., Liu, Y., Dell'Anno, M., Sotira, S., et al. (2020). Evaluation of dietary administration of chestnut and quebracho tannins on growth, serum metabolites and fecal parameters of weaned piglets. *Anim. Open Access J. MDPI* 10, 1945. doi: 10.3390/ani10111945

Deng, Z., Wang, J., Wang, J., Yan, Y., Huang, Y., Chen, C., et al. (2024). Tannic acid extracted from gallnut improves intestinal health with regulation of redox homeostasis and gut microbiota of weaned piglets. *Anim. Res. One Health* 2, 16–27. doi: 10.1002/aro2.51

Galassi, G., Mason, F., Rapetti, L., Crovetto, G. M., and Spanghero, M. (2019). Digestibility and metabolic utilisation of diets containing chestnut tannins and their effects on growth and slaughter traits of heavy pigs. *Ital. J. Anim. Sci.* 18, 746–753. doi: 10.1080/1828051X.2019.1570361

Lee, H. J., Choi ,In Hag, Kim ,Dong Hyeon, Amanullah ,Sardar M., and and Kim, S. C. (2016). Nutritional characterization of tannin rich chestnut (*Castanea*) and its meal for pig. *J. Appl. Anim. Res.* 44, 258–262. doi: 10.1080/09712119.2015.1031779

Li, Q. H., Yan, H. S., Li, H. Q., Gao, J. J., and Hao, R. R. (2020). Effects of dietary supplementation with grape seed procyanidins on nutrient utilisation and gut function in weaned piglets. *Anim. Int. J. Anim. Biosci.* 14, 491–498. doi: 10.1017/S1751731119002234

Ma, M., Chambers, J. K., Uchida, K., Ikeda, M., Watanabe, M., Goda, Y., et al. (2021). Effects of supplementation with a quebracho tannin product as an alternative to antibiotics on growth performance, diarrhea, and overall health in early-weaned piglets. *Animals* 11, 3316. doi: 10.3390/ani11113316

Ma, M., Enomoto, Y., Takahashi, T., Uchida, K., Chambers, J. K., Goda, Y., et al. (2024). Study of the effects of condensed tannin additives on the health and growth performance of early-weaned piglets. *Anim. Open Access J. MDPI* 14, 2337. doi: 10.3390/ani14162337

Mariscal-Landín, G., Avellaneda, J. H., Reis de Souza, T. C., Aguilera, A., Borbolla, G. A., and Mar, B. (2004). Effect of tannins in sorghum on amino acid ileal digestibility and on trypsin (E.C.2.4.21.4) and chymotrypsin (E.C.2.4.21.1) activity of growing pigs. *Anim. Feed Sci. Technol.* 117, 245–264. doi: 10.1016/j.anifeedsci.2004.09.001

Miragoli, F., Patrone, V., Prandini, A., Sigolo, S., Dell'Anno, M., Rossi, L., et al. (2021). A mixture of quebracho and chestnut tannins drives butyrate-producing bacteria populations shift in the gut microbiota of weaned piglets. *PloS One* 16, e0250874. doi: 10.1371/journal.pone.0250874

Myrie, S. B., Bertolo, R. F., Sauer, W. C., and Ball, R. O. (2008). Effect of common antinutritive factors and fibrous feedstuffs in pig diets on amino acid digestibilities with special emphasis on threonine. *J. Anim. Sci.* 86, 609–619. doi: 10.2527/jas.2006-793

Ollagnier, C., Mellino, M.-R., Pradervand, N., Tretola, M., Dubois, S., Durosoy, S., et al. (2025). Feed supplementation with potentiated zinc and/or tannin-rich extracts reduces ETEC infection severity and antimicrobial resistance genes in pig. *Front. Vet. Sci.* 12, 1494103. doi: 10.3389/fvets.2025.1494103

Pan, L., Feng, S., Li, W., and Zhu, W. (2022a). Comparative digestion and fermentation characteristics of low-tannin or high-tannin sorghum grain in the porcine gastrointestinal tract. *J. Anim. Sci.* 100, skac300. doi: 10.1093/jas/skac300

Pan, L., Li, W., Gu, X. M., and Zhu, W. Y. (2022b). Comparative ileal digestibility of gross energy and amino acids in low and high tannin sorghum fed to growing pigs. *Anim. Feed Sci. Technol.* 292, 115419. doi: 10.1016/j.anifeedsci.2022.115419

Rajković, E., Schwarz, C., Tischler, D., Schedle, K., Reisinger, N., Emsenhuber, C., et al. (2021). Potential of grape extract in comparison with therapeutic dosage of antibiotics in weaning piglets: effects on performance, digestibility and microbial metabolites of the ileum and colon. *Anim. Open Access J. MDPI* 11, 2771. doi: 10.3390/ani11102771

Schneider, L. I., Borba, A., Medeiros, J. M. D., Klein, D. R., Poletti, B., Rossi, C. A. R., et al. (2024). Black wattle (*Acacia mearnsii*) condensed tannin extract as feed additive in diets of weaned piglets. *Ciênc. Rural* 54. doi: 10.1590/0103-8478cr20220515

Seoni, E., Battacone, G., Ampuero Kragten, S., Dohme-Meier, F., and Bee, G. (2021). Impact of increasing levels of condensed tannins from sainfoin in the grower-finisher diets of entire male pigs on growth performance, carcass characteristics, and meat quality. *Anim. Int. J. Anim. Biosci.* 15, 100110. doi: 10.1016/j.animal.2020.100110

Song, Y., Luo, Y., Yu, B., He, J., Zheng, P., Mao, X., et al. (2021). Tannic acid extracted from gallnut prevents post-weaning diarrhea and improves intestinal health of weaned piglets. *Anim. Nutr.* 7, 1078–1086. doi: 10.1016/j.aninu.2021.04.005

Souza, K. L. de, Dias, C. P., Callegari, M. A., Friderichs, A., Paes, A. O. S., de Carvalho, R. H., et al. (2025). Performance and intestinal health of piglets in the nursery phase subjected to diets with condensed black wattle (*Acacia mearnsii*) tannin. *Anim. Biosci.* 38, 117–130. doi: 10.5713/ab.24.0112

Steendam, C. A. C., Tamminga, S., Boer, H., de Jong, E.-J., Visser, G. H., and Verstegen, M. W. A. (2004a). Ileal endogenous nitrogen recovery is increased and its amino acid pattern is altered in pigs fed quebracho extract. *J. Nutr.* 134, 3076–3082. doi: 10.1093/jn/134.11.3076

Steendam, C. A. C., Verstegen, M. W. A., Tamminga, S., Boer, H., van 't End, M., Verstappen, B., et al. (2004b). Route of tracer administration does not affect ileal endogenous nitrogen recovery measured with the 15N-isotope dilution technique in pigs fed rapidly digestible diets. *J. Nutr.* 134, 3068–3075. doi: 10.1093/jn/134.11.3068

Verhelst, R., Schroyen, M., Buys, N., and Niewold, T. (2014). Dietary polyphenols reduce diarrhea in enterotoxigenic *Escherichia coli* (ETEC) infected post-weaning piglets. *Livest. Sci.* 160, 138–140. doi: 10.1016/j.livsci.2013.11.026

Xu, T., Ma, X., Zhou, X., Qian, M., Yang, Z., Cao, P., et al. (2022). Coated tannin supplementation improves growth performance, nutrients digestibility, and intestinal function in weaned piglets. *J. Anim. Sci.* 100, skac088. doi: 10.1093/jas/skac088

Yi, H., Wang, Z., Yang, B., Yang, X., Gao, K., Xiong, Y., et al. (2023). Effects of zinc oxide and condensed tannins on the growth performance and intestinal health of weaned piglets in ETEC-challenged environment. *Front. Microbiol.* 14, 1181519. doi: 10.3389/fmicb.2023.1181519

Zhang, Q., Zhang, L., Du, L., Zhang, Y., Yi, D., Zhao, D., et al. (2023). Dietary supplementation of natural tannin relieved intestinal injury and oxidative stress in piglets challenged with enterotoxigenic *Escherichia coli*. *Czech J. Anim. Sci.* 68, 296–305. doi: 10.17221/148/2022-CJAS
